# Environmental DNA (eDNA) metabarcoding assays to detect invasive invertebrate species in the Great Lakes

**DOI:** 10.1371/journal.pone.0177643

**Published:** 2017-05-18

**Authors:** Katy E. Klymus, Nathaniel T. Marshall, Carol A. Stepien

**Affiliations:** Great Lakes Genetics/Genomics Laboratory, Department of Environmental Sciences, University of Toledo, Toledo, OH, United States of America; University of Hyogo, JAPAN

## Abstract

Describing and monitoring biodiversity comprise integral parts of ecosystem management. Recent research coupling metabarcoding and environmental DNA (eDNA) demonstrate that these methods can serve as important tools for surveying biodiversity, while significantly decreasing the time, expense and resources spent on traditional survey methods. The literature emphasizes the importance of genetic marker development, as the markers dictate the applicability, sensitivity and resolution ability of an eDNA assay. The present study developed two metabarcoding eDNA assays using the mtDNA 16S RNA gene with Illumina MiSeq platform to detect invertebrate fauna in the Laurentian Great Lakes and surrounding waterways, with a focus for use on invasive bivalve and gastropod species monitoring. We employed careful primer design and *in vitro* testing with mock communities to assess ability of the markers to amplify and sequence targeted species DNA, while retaining rank abundance information. In our mock communities, read abundances reflected the initial input abundance, with regressions having significant slopes (p<0.05) and high coefficients of determination (R^2^) for all comparisons. Tests on field environmental samples revealed similar ability of our markers to measure relative abundance. Due to the limited reference sequence data available for these invertebrate species, care must be taken when analyzing results and identifying sequence reads to species level. These markers extend eDNA metabarcoding research for molluscs and appear relevant to other invertebrate taxa, such as rotifers and bryozoans. Furthermore, the sphaeriid mussel assay is group-specific, exclusively amplifying bivalves in the Sphaeridae family and providing species-level identification. Our assays provide useful tools for managers and conservation scientists, facilitating early detection of invasive species as well as improving resolution of mollusc diversity.

## Introduction

Detecting and monitoring species presence using environmental DNA (eDNA) is a rapidly growing research area. Species identification from recovered DNA that was shed into the environment by organisms constitutes a powerful tool for conservation biologists, allowing them to monitor taxa with greater sensitivity, less effort, and fewer negative affects relative to traditional survey methods [1–2]. Since only trace DNA amounts are required, it is particularly attractive to detect low abundance taxa. Consequently, there is great interest in using eDNA tools for early detection of invasive species when populations are small and confined, which can facilitate successful eradication outcomes [3]. Most initial eDNA studies have taken a targeted single species-specific approach, using conventional PCR, quantitative PCR (qPCR) or digital droplet PCR [4–6]. Others have used this single-species approach to investigate eDNA production and degradation dynamics [7–10].

Alternative to the single species-specific approach, analyzing eDNA with high-throughput (also termed next-generation) sequencing allows for multiple taxa identification simultaneously from a single sample via the general concepts of DNA barcoding [11] and metabarcoding [12]. Metabarcoding is especially useful for discerning unanticipated taxa. Beyond species detection, metabarcoding further enables the analysis of community composition from eDNA samples [13–17]. Although promising, difficulties in applying metabarcoding techniques to eDNA samples exist, such as obtaining species level discriminatory markers, as well as possible PCR and sequencing biases. The latter may influence the presumed relationship between sequence reads and eDNA abundance.

Metabarcoding studies rely on universal primers that amplify DNA of multiple target species at a specific locus. For samples that contain the entire organism (e.g., microorganisms, meiofauna, bulk samples, diet analyses, eggs, gametes, spores, or larvae), universal primers that target a large barcoding region (i.e., COI in animals [11], rbcL and matK in plants [18], and ITS in fungi [19]) have been used [20–22]. However, macro-organismal DNA that has been cast into the environment (e.g., eDNA, forensic sampling) often is degraded, which results in poor amplification of larger markers [23]. Thus a difficulty in eDNA metabarcoding is finding short amplifiable markers (~100–200 base pairs) that can discriminate among species. Such mini-barcodes have been developed for some taxa; for instance, Hajibabei *et al*. [24] successfully amplified and identified museum arthropod specimens using markers 100–400 bp long. For primer development, researchers either have used programs such as EcoPrimer [25] and Primer Tree [16], or visually discerned prospective markers. Despite these efforts, it remains difficult to target species-discrimination regions given the short length eDNA marker requirements.

A second concern related to metabarcoding of eDNA, is whether read abundance can be used to assess a species’ abundance or biomass [26]. Targeted species specific studies using qPCR have found positive relationships between copy number and species biomass [7,27]; however, few eDNA metabarcoding studies have reported a similar relationship between read abundance and biomass, and those that have, discerned moderate rather than strong relationships [14,17]. Furthermore many studies assume that high-throughput sequencing produces quantitative output, but do not explicitly test whether read abundance accurately reflects the starting amount of DNA. Notably, metabarcoding of bulk samples [28–29] and artificial communities of fungal PCR products [30] indicate that biases in primer binding, sequencing, and bioinformatic pipelines can limit quantitative metabarcoding abilities.

To address these two issues, we designed high-throughput sequencing assays to discern invasive mollusc species in the Laurentian Great Lakes by developing group-specific (rather than broadly universal) primers and tested them using mock communities of mixed template samples, at various proportions of concentrations. Focusing on potential primer bias, the group-specific primers were formulated to detect 19 invasive or potentially invasive bivalve and snail species listed on government databases and watch lists [31–33]. These included NOAA’s Great Lakes Aquatic Nonindigenous Species Information System (GLANSIS) database [31], the U.S. Army Corps of Engineers Great Lakes and Mississippi River Interbasin Study (GLMRIS) report “*Non-native Species of Concern and Dispersal Risk for the Great Lakes and Mississippi River Interbasin Study*” [32], and the USEPA report “*Predicting Future Introductions of Nonindigenous Species to the Great Lakes*” [33]. We also developed a primer set specific to the Sphaeriidae family of clams, a cosmopolitan group having several native and invasive species in the Great Lakes region. Like other understudied invertebrates, their identification requires extensive taxonomic expertise due to morphological similarity and phenotypic plasticity. Our goal was to develop primers that amplify short (100–300 bp) fragments to discriminate among species, aiding taxonomic studies and surveys. To address PCR amplification bias, we used mock communities containing known amounts of DNA (copy number) per species to validate our assays. Finally, the assays were used to test eDNA samples from known lab aquarium assemblages and the field.

## Materials and methods

### Primer design and reference database development

We evaluated three DNA regions that have been used in phylogenetic studies of the targeted invertebrate taxa. Sequences from 16S mitochondrial (mt) ribosomal (r)DNA, cytochrome oxidase I (COI) mtDNA, and 28S nuclear rDNA genes were downloaded from NIH GenBank (https://www.ncbi.nlm.nih.gov/genbank/) for all targeted species. Relative to the COI and 28S data, we found that the 16S sequence data were most abundant and well-resolved for the targeted taxa. Furthermore, 16S was found to possess conserved regions in which primers could readily be designed. Those regions were interspersed with interspecific variable regions, allowing for targeted species identification. COI appeared too variable, leading to difficulty in primer design and 28S was too conserved, circumventing discrimination among species. Focusing on 16S, we used the downloaded GenBank 16S sequences for the targeted taxa to design out reference database (Table 1). Specimens of these taxa and close relatives had their DNA extracted, amplified, and sequenced at the 16S locus using universal 16S primers, 16Sar-L: 5’-CGCCTGTTTATCAAAAACAT-3’ and 16Sbr-H: 5’-CCGGTCTGAACTCAGATCACGT-3’ [34]. Polymerase chain reactions (PCR) of 25 μl, including 16.7 μl ddH_2_O, 1X AmpliTaq® PCR buffer, 0.8 mM dNTPs, 0.5 μM of each primer, 1.5 U AmpliTaq®, and 25–50 ng template DNA. Cycling conditions consisted of initial denaturation at 94° C for 5 min, followed by 40 cycles of 94° C for 1 min, 48.5°C for 1 min, and 72°C for 1.5 min, with a final 5 min extension at 72°C. PCR products were purified using QIAquick^®^ PCR Purification kits (Qiagen). Sequencing was performed at Cornell University Life Sciences Core Laboratories Center using an Applied Biosystems Automated 3730 DNA Analyzer (Fullerton, CA, USA). These sequences then were added to the reference database of downloaded GenBank (https://www.ncbi.nlm.nih.gov/genbank/) sequences in our laboratory (Table 1) as well as to the GenBank nucleotide database (KY426891-KY426915). Subsequent primer design was based on this reference database.

**Table 1:** List of targeted species for the MOL16S assay.

	Species	*Scientific name*	GenBank accession numbers
**Bivalves**	golden mussel	*Limnoperna fortune* (Dunker, 1857)	(1) JQ267790
	*dark false mussel	*Mytilopsis leucophaeata* (Conrad, 1831)	(3) AF507051–52, EF414448 (1) AF038998 (1) KY426891
	*quagga mussel	*Dreissena rostriformis* (Deshayes, 1838)	(2) AF038996, KY426895 (9) DQ333745–46, AF507047–48, AY302247, JX099457, KY426892-94 (1) JQ348913 (1) KY426896
	*zebra mussel	*D*. *polymorpha* (Pallas, 1771)	(14) GBXKY426897-902, DQ333747–48, EF414464–66, AF038997, JX099458, AF507049
	*Asian clam	*Corbicula fluminea* (O. F. Müller, 1774)	(7) AB522656, AF038999, AF152024, DQ280039, KC429294, KY426903-04
	*European fingernail clam	*Sphaerium corneum* (Linnaeus, 1758)	(1) GU128616 (18) GU128617–35 (2) KY426905-06
	^**Ŧ**^grooved fingernail clam	*S*. *similie* (Say, 1817)	(1) KY426907
	greater European peaclam	*Pisidium amnicum* (O. F. Müller, 1774)	(7) EU559086–89, AY093572, DQ062609–10
	henslow peaclam	*P*. *henslowanum* (Sheppard, 1825)	(1) DQ062644 (8) EU559115–22, DQ062620–22 (1) KF483297
	pygmy peaclam	*P*. *moitessierianum* Paladihe, 1866	(3) DQ062626, DQ06262628–29 (1) DQ06262627
	humpbacked peaclam	*P*. *supinum* Schmidt, 1850	(7) DQ062646–50, EU559148, AY093569
	^**Ŧ**^ridged-beak peaclam	*P*. *compressum* Prime, 1852	(1) AY093560 (2) AF152029, KY426908 (1) AY957810 (1) AY957812
**Gastropods**	*New Zealand mud snail	*Potamopyrgus antipodarum* (Gray, 1843)	(1) AY634079 (1) AY955392 (2) AY9553886–87 (3) AY955388–89, AY634106 (1) AY955377 (1) AY955378 (1) AY955381 (1) AY955391 (8) AY955379–80, AY955382–85, AY634104, AY634107 (5) JN639013–14, KY426909, JQ346706, AY955393 (1) AY634109 (10) JQ346702–05, JQ346708–09, AY634080, AY955376, AY314009, EU573989
	*faucet snail	*Bithynia tentaculata* (Linnaeus, 1758)	(2) AF445344, JX970531 (1) FJ160288
	*Oriental mystery snail	*Cipangopaludina chinensis malleata/ japonica* (Gray, 1834)	*C*. *chinensis*: (2) FJ710213–214 (8) LC028474–76, LC028479–81, KY42610-11, (1) LC028482 (4) LC0284732, LC028483–84, LC028477 (1) LC028473 (1) LC028478; *C*. *japonica*: (1) LC028460 (1) LC028461 (1) LC028463 (5) LC028462, LC028464–66, LC028468 (1) LC028467 (1) LC028469 (2) LC028470–71 (1) FJ405736; *C*. *diachiensi*s: (1) GU1988839 (3) GU198835, GU198871–72 (1) GU198836; *C*. *longispira*: (2) GU198863–64 (1) KJ867106; *C*. *ventricosa*: (1) KJ867107
	*buffalo pebble snail	*Gillia altilis* (Lea, 1841)	(1) KY426912
	*red-rim melania (Malaysian trumpet snail)	*Melanoides tuberculata* (O. F. Müller, 1774)	(1) AF101006 (1) KP284119 (1) KP284124 (5) KP284118, KP284120, KP284122, KP2841227, AY010517 (9) KP774656–58, KP774669–72, AY791930–31 (23) AY791911, AY791915, KP774640, KP774644–45, KP774647–55, KP774659, KP774661–62, KP774664–68, KY426914 (1) KP284121 (1) AY456618 (5) AY791910, KP774660, KP774663, AY456616–17 (1) KP284126 (1) AY791914 (7) KP774633–39 (3) KP284125, KP284128–29
	island apple snail	*Pomacea maculata Syn*. *P*. *insularum* Perry, 1810	*P*. *insularum* (1) EF519108 (1) FJ71028 (1) FJ71029; *P*. *bridgesi*: (2) EU274500, KC109970; *P*. *papyrace*a: (1) FJ710248; *P*. *guyanensis*: (1) FJ710243; *P*. *flagellata*: (1) FJ710247; *P*. *patula*: (1) FJ710246; *P*. *sordida*: (1) FJ710244; *P*. *camena*: (1) FJ710245; *P*. *haustrum*: (1) FJ710239; *P*. *paludos*a: (1) FJ710237 (1) FJ710238; *P*. *doloides*: (1) FJ710232 (1) FJ710233; *P*. *lineata*: (1) FJ710230 (1) FJ710231; *P*. *diffusa*: (1) FJ710242 (1) KM389472; *P*. *scalaris*: (1) FJ710240 (1) FJ710241; *P*. *canaliculata*: (1) KF032562 (4) KF032563–65, KF032578 (1) KF032570 (1) KF032572 (1) KF032574 (1) KF032577 (1) FJ710236 (1) FJ710234 (9) KF032500-501, KF032503-504, KF032507, KF032566–67, KF032569, KF032579 (1) KF032568 (1) KF032571 (1) KF032573 (1) KF032575 (1) KF032576 (7) EU27450, FJ710235, KF002499, KF002502, KF002505–06, KJ766112
	European ear snail	*Radix auricularia* (Linnaeus, 1758)	*R*. *auricularia*: (1) AF485646 (2) KP098540, NC_026538; *R*. *peregra*: (2) HQ283242, U82074; *R*. *rubiginos*a: (2) GU 451749, GU167907 (1) U82076; *R*. *natalenisis*: (1) HQ28342; *Radix balthica: (1) KY426913
	*European stream valvata	*Valvata piscinalis* (O. F. Müller, 1774)	(1) FJ917248 (1) KY426915
	banded mystery snail	*Viviparus georgianus* (Lea, 1834)	(1) AY377626

Parentheses denote unique OTUs for the MOL16S amplicon, with the number inside representing the number of sequences belonging to that OTU. Accession numbers are for 16S sequence data for each species.

* indicates invasive species whose extractions were used to test primers *in vitro*.

**Ŧ** indicates native or non-invasive species also used to test primers. All other species are invasive in the Great Lakes.

Sequences first were aligned using Geneious Software (7.1.9) with MUSCLE [35]. Alignments were verified by visual inspection and corrections were made. Non-target taxa (human *Homo sapiens*, walleye *Sander vitreus*, and spiny water flea *Bythotrephes longimanus*) also were included in alignments to assess the relative target specificity of the primers. Primer pairs were designed by visually inspecting alignments and searching for regions of diagnostic interspecific variability in the target taxa that were flanked by conserved regions. Primers then were evaluated using IDT’s OligoAnalyzer ® software for GC content, annealing temperature, and possible dimer products. Finally, primer pairs were tested *in vitro* with DNA extractions from targeted species for which we had tissue samples from (Table 1), including a non-target fish species (*S*. *vitreus*).

We developed primer sets for two assays. First, degenerate primers were designed to differentiate invasive bivalve and snail species using a short fragment of the mtDNA 16S RNA gene (herein referred to as MOL16S assay): MOL16S_F: 5’–RRWRGACRAGAAGACCCT– 3’, MOL16S_R: 5’-ARTCCAACATCGAGGT-3’. The primer set amplified some non-target species as well, since it is partially conserved across other invertebrate taxa and some fishes. Due to concern that fish DNA in an environmental water sample could potentially swamp out invertebrate DNA signal, a blocking primer was designed to reduce the former. The 5’ end of the blocking primer overlaps the 3’ MOL16S reverse primer, but extends further to capture fish specific nucleotide variation to increase specific binding to fish DNA. The 3’ end was modified with a C3 spacer to prevent product elongation [36], MOL16S_FISBLOCK: 5’–AGGTCGTAACCCCCTRG/3SpC3/–3’. For our second assay, non-degenerate primers amplified all 58 identified sphaeriid mussel species obtained from GenBank sequences for 16S (herein referred to as SPH16S assay) (S1 Table), SPH16S_F:5’–TAGGGGAAGGTATGAATGGTTTG–3’, SPH16S_R: 5’–ACATCGAGGTCGCAACC–3’. Sphaeriid mussels are defined here as the three genera in the Sphaeriidae family (*Sphaerium*, *Musculium*, *Pisidium*,and *Euglesa*). Targeted amplicon length for SPH16S was 299 bp, whereas amplicon length varied among species for the MOL16S assay from 183–310 bp.

### Mock community design

Five mock communities were designed to encompass varying copy numbers of the targeted amplicon from 11 species (Table 2). We included two native sphaeriid species, as well as walleye, to increase diversity and to test the fish blocking primer. To quantify the target copy number per extraction, a series of competitive PCRs were run, in which an extraction or native template (NT) and an internal standard (IS) were co-amplified with the primer set. The IS is a synthetic double stranded homolog of the target marker that has the same primer annealing sites as the NT, enabling it to compete equally for the same primers, as well as a small deletion that enables products of the IS and NT to be differentiated by size. Given the similar amplification efficiencies between IS and NT, the initial IS and NT proportions should correspond to their ratio in the amplified product [37]. Using known concentrations (copy number/μl) of IS, we determined when this ratio was 1:1 and calculated the NT copy number. To determine this 1:1 ratio, a set of PCRs with a constant amount of target DNA extraction (25 ng) and a known IS amount were amplified, with the latter serially diluted among reactions. Molarity of each product was measured on a 2100 Bioanalyzer (Agilent Technologies). We then calculated the ratios of the log10 transformed NT:IS template molarities, which were regressed against the log10 transformed copy number of known IS concentrations used in each competitive PCR. The y-intercept of the regression line indicated the 1:1 ratio of NT:IS, which was the copy number concentration of the NT. Once copy number concentrations of the original DNA extractions were determined, we created our mock community samples using calculated volumes of the extraction required to provide the desired copy number for each target species.

**Table 2:** Composition of the five mock communities showing the target amplicon copy number per extraction.

Species	Mock Communities				
	1	2	3	4	5
*Sphaerium similie*	9090	1818	363	72	14
*Dreissena rostriformis*	1818	363	72	14	9090
*Sander vitreus* (walleye)	363	72	14	9090	1818
*Sphaerium corneum*	72	14	9090	1818	363
*Pisidium compressum*	14	9090	1818	363	72
*Mytilopsis leucophaeata*	2272	1136	568	284	142
*Dreissena polymorpha*	1136	568	284	142	2272
*Potamopyrgus antipodarum*	568	284	142	2272	1136
*Gillia altilis*	284	142	2272	1136	568
*Cipangopaludina chinensis*	142	2272	1136	568	284
*Melanoides tuberculata*	18	18	18	18	18

To distinguish IS and NT amplification products on the 2100 Bioanalyzer (Agilent Technologies), IS was ordered as a gBlocks ® gene fragment (IDT) of target amplicons with a deletion interior to the priming sites, making the IS 10% shorter than the NT amplicon. A 10% difference in size has been shown to not cause differences in PCR efficiency between NT and IS templates [36].

### eDNA samples from aquaria and the Maumee River

We collected *Corbicula fluminea*, *Dreissena polymorpha*, *D*. *rostriformis*, sphaeriid clams (*Sphaerium* sp. and *Pisidium* sp.) and pleurocerid snails from streams in western Lake Erie during summer 2016. The sphaeriid clams were morphologically identified to genus level and the snails were identified to family level. These were placed in two 38-liter aquaria, whose assemblage compositions are described in Table 3. After two weeks, one liter of water was collected from each aquarium (the water was first mixed) for eDNA extraction and analysis.

**Table 3:** Number of individuals from each species placed into two aquaria for later eDNA sampling.

Species	Approximate *N* individuals Tank A	Approximate *N* individuals Tank B
*Sphaerium spp*.	40	40
*Pisidium spp*.	10	0
*Dreissena spp*.	10	10
*Corbicula fluminea*	4	1
Pleurocerid snails	3	3

The Ohio EPA gathered data for identifying fish and macroinvertebrate assemblages using traditional sampling methods in the Maumee River in 2012. At the same time, they took one liter water samples from each site for later eDNA analysis by the Stepien laboratory, which were labeled and stored at -80°C, and used here.

### Samples and DNA extractions

Extractions for the mock community samples and primer testing were prepared using tissue from taxonomically identified specimens stored in 95% EtOH (S2 & S3 Tables). Specimens were identified by the original collector (S3 Table). The Qiagen DNeasy® Blood and Tissue kit was used to extract bivalve DNA, whereas the Omega Bio-tek E.Z.N.A® Mollusc DNA Kit was used for snail DNA.

Water samples were filtered through a polyethersulfone (PES) membrane using a vacuum pump. For the aquarium samples, one liter of water was filtered through a 0.45-micron PES filter, and 400 ml of the river water samples were filtered with a 0.2-micron PES filter. We also filtered and extracted DNA from 400 ml of molecular grade purified ddH2O as a negative control or blank sample. DNA from water samples was extracted following the Turner *et al*. [38] cetyl trimethyl ammonium bromide (CTAB) protocol. Three river water samples and the blank were tested with our MOL16S assay (without the fish blocking primer). Sample selection was based on presence of invasive species from our list, according to Ohio EPA’s visual identification data (http://www.epa.ohio.gov/Portals/35/documents/MaumeeTSD_2014.pdf) [39]. Aquaria samples were tested with both the MOL16S (without the fish blocking primer) and the SPH16S assays.

### Library preparation

To prepare samples for paired-end, high-throughput sequencing on the MiSeq platform (Illumina, San Diego, CA, USA), libraries were prepared in a two step PCR process. In the first step, the target was amplified with assay specific primers that were appended with 7–17 additional nucleotides (spacers) and a 33–34 bp sequencing primer region on the 5’ end. Four primer sets were created, which differed only in their spacer combinations (Table 4). The spacer region increased nucleotide diversity of the sequence reads and enhanced cluster formation for improved sequencing on the MiSeq platform [40–41] and was not analogous to the C3 spacer modification used in the blocking primer. Each sample was prepared with one of these four primer pairs. PCRs contained 25 μl reaction volume, including 14.69 μl of the 10 μM blocking primer (5.88 μM final concentration), 1X NEB PCR buffer, 0.2 mM dNTPs, 0.5 μM of each primer, 1.57 U of AmpliTaq ®, and 2.5 μl of template DNA. In samples lacking the fish blocking primer, the volume of blocking primer was replaced with an equal volume of ddH_2_0. Conditions were: 30 sec initial denaturation at 95°C, followed by 25–35 cycles of 95°C for 30 sec, 58°C for 30 sec, and 68°C for 1 min, with a 2 min final extension at 68°C. Products were cleaned using QIAquick® PCR purification kits (Qiagen).

**Table 4:** Primers used for library preparation.

Assay	Primers	Sequence 5’– 3’
MOL16S	MOL16S_E_F	*TCGTCGGCAGCGTCAGATGTGTATAAGAGACAG*TCCTATG**RRWRGACRAGAAGACCCT**
	MOL16S_F_F	*TCGTCGGCAGCGTCAGATGTGTATAAGAGACAG*ATGCTACAGT**RRWRGACRAGAAGACCCT**
	MOL16S_G_F	*TCGTCGGCAGCGTCAGATGTGTATAAGAGACAG*CGAGGCTACAACTC**RRWRGACRAGAAGACCCT**
	MOL16S_H_F	*TCGTCGGCAGCGTCAGATGTGTATAAGAGACAG*GATACGATCTCGCACTC**RRWRGACRAGAAGACCCT**
	MOL16S_E_R	*GTCTCGTGGGCTCGGAGATGTGTATAAGAGACAG*CGTACTAGATGTACGA**ARTCCAACATCGAGGT**
	MOL16S_F_R	*GTCTCGTGGGCTCGGAGATGTGTATAAGAGACAG*TCACTAGCTGACGC**ARTCCAACATCGAGGT**
	MOL16S_G_R	*GTCTCGTGGGCTCGGAGATGTGTATAAGAGACAG*GAGTAGCTGA**ARTCCAACATCGAGGT**
	MOL16S_H_R	*GTCTCGTGGGCTCGGAGATGTGTATAAGAGACAG*ATCGGCT**ARTCCAACATCGAGGT**
SPH16S	SPH16S_E_F	*TCGTCGGCAGCGTCAGATGTGTATAAGAGACAG*TCCTATG**TAGGGGAAGGTATGAATGGTTTG**
	SPH16S_F_F	*TCGTCGGCAGCGTCAGATGTGTATAAGAGACAG*ATGCTACAGT**TAGGGGAAGGTATGAATGGTTTG**
	SPH16S_G_F	*TCGTCGGCAGCGTCAGATGTGTATAAGAGACAG*CGAGGCTACAACTC**TAGGGGAAGGTATGAATGGTTTG**
	SPH16S_H_F	*TCGTCGGCAGCGTCAGATGTGTATAAGAGACAG*GATACGATCTCGCACTC**TAGGGGAAGGTATGAATGGTTTG**
	SPH16S_E_R	*GTCTCGTGGGCTCGGAGATGTGTATAAGAGACAG*CGTACTAGATGTACGA**ACATCGAGGTCGCAACC**
	SPH16S_F_R	*GTCTCGTGGGCTCGGAGATGTGTATAAGAGACAG*TCACTAGCTGACGC**ACATCGAGGTCGCAACC**
	SPH16S_G_R	*GTCTCGTGGGCTCGGAGATGTGTATAAGAGACAG*GAGTAGCTGA**ACATCGAGGTCGCAACC**
	SPH16S_H_R	*GTCTCGTGGGCTCGGAGATGTGTATAAGAGACAG*ATCGGCT**ACATCGAGGTCGCAACC**

Assay specific primers (in bold) with a 7–17 nucleotide spacer (underlined) and the sequencing primer region (italicized). 5’ to 3’ direction for all primers, the last character in the primer name indicates direction (F = forward, R = reverse).

The second PCR step used the prior step’s column-cleaned product as the template and incorporated Nextera paired end indices (Illumina ®, kit FC-121-1011) as well as the P5 and P7 adaptor sequences, which enabled the prepared product to bind onto the surface of the Illumina® MiSeq flowcell. The added indices allowed for multiple samples to be pooled together in a single MiSeq run. PCR was carried out in a 25 μl reaction including a variable volume of H_2_0 (depending on volume of template added), 1X NEB (New England Biolabs) PCR buffer, 0.2 mM dNTPs, 2.5 μl of each indexed primer, 1.57 U of NEB Hotstart Taq polymerase, and up to 24 ng of purified product from the first reaction. Conditions consisted of a 30 sec initial denaturation at 95°C, followed by 8 cycles of 95°C for 30 sec, 55°C for 30 sec, and 68° C for 1 min, with a final 2 min extension at 68°C.

After column clean up, product was sized and quantified using a 2100 Bioanalyzer (Agilent Technologies) before sending to Ohio State University’s Molecular and Cellular Imaging Center in Wooster, OH for MiSeq analysis (http://mcic.osu.edu/). To avoid sequencing dimer product observed around 200–250 bp, the targeted fragments (320–550 bp) were size-selected with a 1.5% agarose gel cassette on Pippin Prep (Sage Science). Concentration of pooled product was measured with a Qubit fluorometer (Invitrogen). Pooled samples were run on an Illumina® MiSeq with 2 X 300 bp V3 chemistry. An additional 40–50% PhiX DNA spike-in control was added to improve data quality of low nucleotide diversity samples.

The first MiSeq run included the following 10 pooled samples: (1–5) one sample from each mock community with the MOL16S primers and fish blocking primer, (6–7) samples from mock communities 3 and 4 using MOL16S primers but without the fish blocking primer, and (8–10) samples from mock communities 1, 2, and 4 with the SPH16S primers. Two additional MiSeq runs were conducted to sequence the water samples.

### Bioinformatics analyses

Reads returned from sequencing were trimmed of adaptors and sequencing primers. We used custom PERL scripts to merge paired reads and trim assay-specific primers [42]. For the mock community samples, an exhaustive search was implemented via custom PERL script to assign reads to taxa using the reference sequence database. For the mock community sample, only reads that were an exact match were retained, thus eliminating chimeric sequences and sequencing errors. For the water samples, our custom reference sequence datasets were not used to identify reads. Instead, after primer trimming, reads were clustered using QIIME’s pick_de_novo_otus python workflow script with the default 97% similarity threshold and UCLUST [43–44]. The subsequent output file, OTU Biom (Biological Observation Matrix; http://biom-format.org/) table, and representative sequence set then were employed to create a table listing the number of reads that matched each OTU (operational taxonomic unit) with the Biom package in R [45–46]. Finally, a BLAST [47] search was performed on the OTU frequency table using the NCBI nucleotide database to identify each OTU to species. The search retained each OTU’s first BLAST hit sequence, scientific name, accession number, identity percentage, coverage percent, and e- (expect) value. Only OTUs with ≥80% coverage and more than one sequence were retained. Reads that were assigned the same GenBank accession number were grouped together as a single OTU. OTUs assigned an identity (% similarity between query and subject sequence) ≥97% were considered identified to species level.

### Statistical analyses of mock communities

#### MOL16S assay

Percentages of reads per species in each mock community were calculated, log10 transformed, and analyzed by regressing observed on to expected read percentages. We compared these within species (among mock communities), as well as within mock communities (among species).

To compare the ability of the blocking primer to reduce amplification of walleye (and other fish) DNA, we calculated the reduction in walleye DNA amplification in the two mock community samples run with and without the blocking primer.

#### SPH16S assay

Since just three sphaeriid species were included in the mock communities, we did not run regression analyses on those results. Instead we show the untransformed expected and observed read percentages for comparison.

#### eDNA samples

To compare our DNA sequence results from the aquaria and river water samples with those from their morphological identifications, we included data from both the molecular and the morphological analyses that were defined at species level. For the molecular barcoding assays, these included those OTUs having 80% coverage and 97% similarity to query sequences from the BLAST search (as discussed above). For the morphological identifications, those taxa that were defined at the species level by morphology were included (S1 File).

## Results

### Primers and amplification results

According to our reference database, the MOL16S and SPH16S primer sets should amplify all targeted species (see Table 1). Primers were tested *in vitro* using tissue extractions (*labels, Table 1), which included three non-targeted species: two native sphaeriid mussels *Sphaerium similie* and *Pisidium compressum*, and the snail *Radix balthica*, which is a congeneric relative of the targeted European ear snail (*R*. *auricularia*) for which we did not have tissue. All extractions amplified with MOL16S. Just the Sphaeriid mussels amplified with the SPH16S primers, indicating their targeted specificity.

### MiSeq read results

Sequencing of the pooled libraries from our first run resulted in a total of 15,184,208 reads that passed cluster quality filtering. Of these, 53% were indexed and corresponded to our samples. This is in concordance with the 40–50% PhiX spike in. Of the indexed reads, approximately 84% merged using the custom PERL script, leading to 6,790,019 merged reads. Primers then were trimmed and taxa assigned to reads using custom PERL scripts and an exhaustive search with our reference sequence database. Percentage of reads that were trimmed ranged from 83.44–99.89% among our 10 samples, and the trimmed reads that exactly matched the database sequences ranged from 25.83–50.08% (S4 Table). The eDNA samples were divided between two other MiSeq runs. These two runs included a number of samples that were part of another study. The first run, which included two Ohio EPA water samples and the water blank, had 15,209,380 reads that passed quality filtering. Of these, 58% (8,821,442) reads were indexed and the rest belonged to the ~40–50% PhiX spike in. Using our custom PERL script, 95–97% merged correctly (excluding the blank sample in which only 5% of reads merged). Finally, 53–95% of these reads were retained after processing, which included: trimming primers, removing singletons, and excluding reads <100 bp long. The second run included one additional Ohio EPA water sample and the four aquaria samples. In this run, 14,329,380 reads passed quality filtering and 41% (5,857,044) were indexed for our samples, with the rest attributed to the PhiX spike. Using our custom PERL script, 96–98% of the reads merged correctly. Finally, the percentage of reads that remained after primer trimming and length processing ranged from 50–96% (S5 Table).

### MOL16S assay mock communities

**Fig 1:**
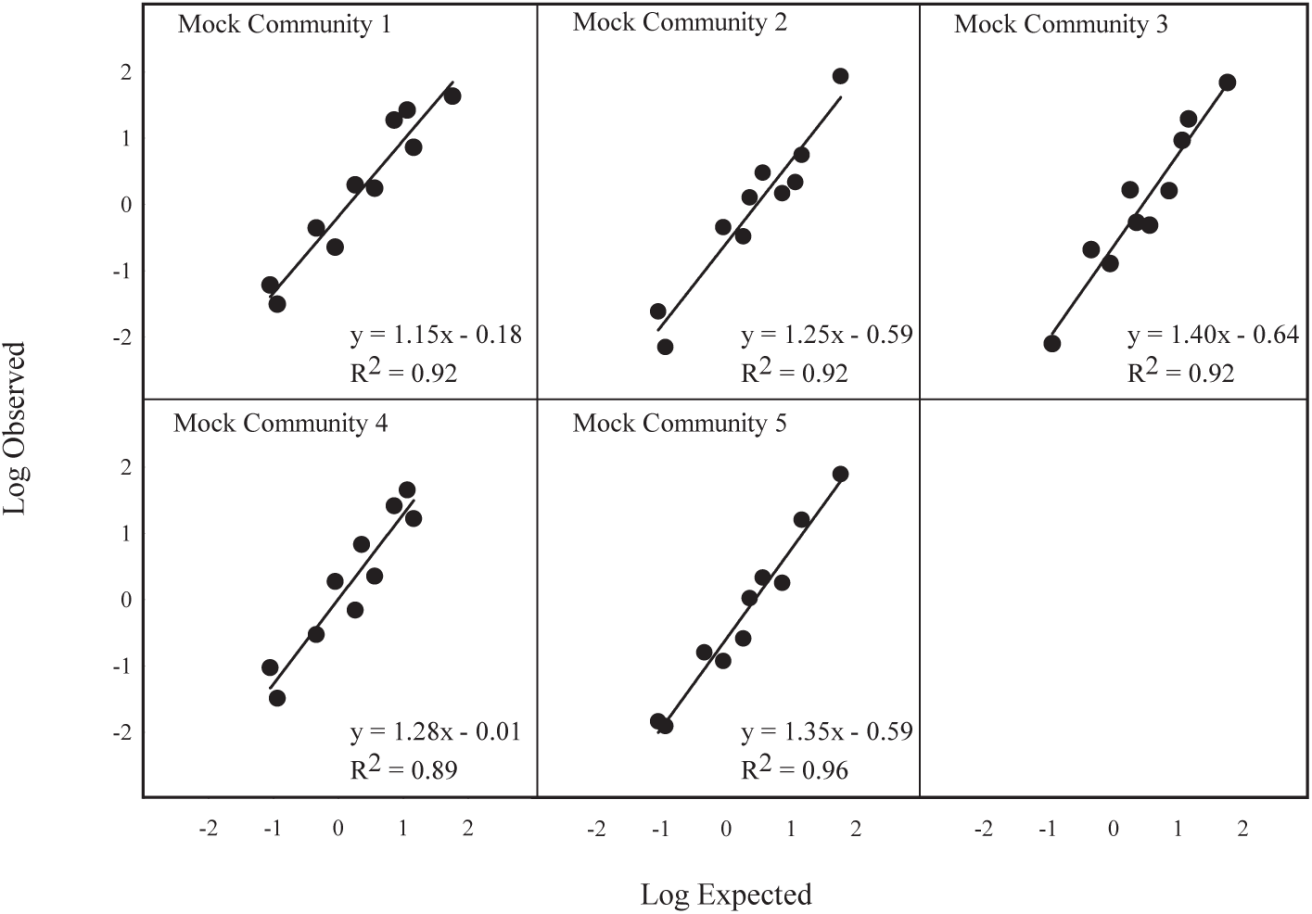
Regressions of log 10 transformed observed read percentages versus log 10 transformed expected read percentages for each of the five mock communities run with the MOL16S assay.

**Fig 2:**
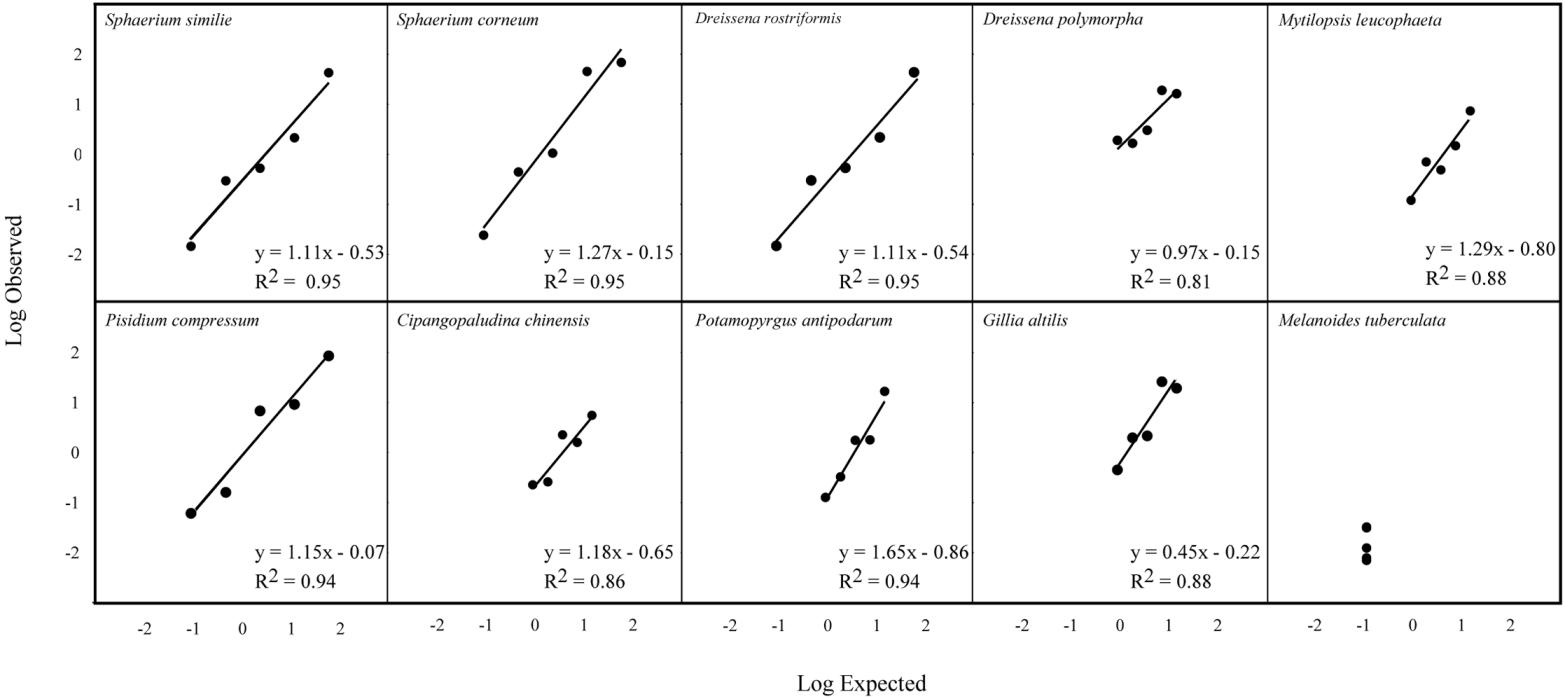
Regressions of log 10 transformed observed read percentages versus log 10 transformed expected read percentages for each of the species in the mock communities run with the MOL16S assay.

For each sample, the total number of reads, percentage of expected reads, and percentage of observed reads per species are shown in Table 5. All regression comparisons revealed significant relationships (*p*<0.05) between observed and expected percentage of reads for the five mock community samples using the MOL16S and fish blocking primers (Figs 1 and 2), indicating that the observed read percentages closely reflected those expected. The two samples without fish blocking primer (replicate samples of mock communities 3 and 4) showed an 88–100% reduction in amplified walleye DNA (Table 6), while yielding similar proportions of reads for the other species. Since the proportion of walleye reads was smaller than expected for all mock communities even without the fish blocking primer, we believe that the extraction copy number was miscalculated leading to a lower than expected copy numbers of walleye in each mock community. Because of this, we also include results comparing the use of the fish blocking primer in a preliminary data set from a separate mock community composition. Those results show that even at relatively high concentrations of fish DNA (~41% sample 1), we obtained a 62% reduction in walleye DNA with the blocking primer (Table 6).

**Table 5:** Number of reads, percentage observed and percentage expected for each of the five mock community samples run with the MOL16S assay and fish blocking primer.

	**Mock Community 1**			**Mock Community 2**			**Mock Community 3**		
**Species**	**Read Number**	**% Observed**	**% Expected**	**Read Number**	**% Observed**	**% Expected**	**Read Number**	**% Observed**	**% Expected**
*Sphaerium similie*	138075	43.11	57.62	3745	2.15	11.52	885	0.53	2.30
*Dreissena rostriformis*	85010	26.54	11.52	2174	1.25	2.30	341	0.20	0.46
*Sander vitreus* (walleye)	13	0.00	2.30	0	0.00	0.46	0	0.00	0.09
*Sphaerium corneum*	1385	0.43	0.46	42	0.02	0.09	112573	67.28	57.62
*Pisidium compressum*	195	0.06	0.09	149359	85.89	57.62	15359	9.18	11.52
*Mytilopsis leucophaeta*	23002	7.18	14.40	2536	1.46	7.20	800	0.48	3.60
*Dreissena polymorpha*	59973	18.72	7.20	5166	2.97	3.60	2686	1.61	1.80
*Potamopyrgus antipodarum*	5594	1.75	3.60	565	0.32	1.80	209	0.12	0.90
*Gillia altilis*	6235	1.95	1.80	783	0.45	0.90	31795	19.00	14.40
*Cipangopaludina chinensis*	711	0.22	0.90	9513	5.47	14.40	2654	1.59	7.20
*Melanoides tuberculata*	99	0.03	0.11	12	0.01	0.11	13	0.01	0.11
	**Mock Community 4**			**Mock Community 5**		
**Species**	**Read Number**	**% Observed**	**% Expected**	**Read Number**	**% Observed**	**% Expected**
*Sphaerium similie*	773	0.29	0.46	30	0.01	0.09
*Dreissena rostriformis*	244	0.09	0.09	163825	78.40	57.62
*Sander vitreus* (walleye)	2679	1.02	57.62	63	0.03	11.52
*Sphaerium corneum*	116994	44.64	11.52	2199	1.05	2.30
*Pisidium compressum*	17620	6.72	2.30	331	0.16	0.46
*Mytilopsis leucophaeta*	1794	0.68	1.80	246	0.12	0.90
*Dreissena polymorpha*	4872	1.86	0.90	33459	16.01	14.40
*Potamopyrgus antipodarum*	43807	16.71	14.40	3752	1.80	7.20
*Gillia altilis*	67309	25.68	7.20	4494	2.15	3.60
*Cipangopaludina chinensis*	5921	2.26	3.60	536	0.28	1.80
*Melanoides tuberculata*	84	0.03	0.11	26	0.01	0.11

**Table 6:** Number of reads, percentage observed and percentage expected for each of the mock community samples run with and without the fish blocking primer and the MOL16S assay.

	**Mock Community 3**				**Mock Community 4**			
**Species**	**Read Number with FB**	**% Observed with FB**	**Read Number without FB**	**% Observed without FB**	**Read Number with FB**	**% Observed with FB**	**Read Number without FB**	**% Observed without FB**
*Sphaerium similie*	885	0.529	718	0.496	773	0.295	314	0.229
*Dreissena rostriformis*	341	0.204	284	0.196	244	0.093	257	0.187
*Sander vitreus* (walleye)	0	0.000	2	0.001	2679	1.022	11956	8.717
*Sphaerium corneum*	112573	67.282	100862	69.734	116994	44.638	54766	39.931
*Pisidium compressum*	15359	9.180	12823	8.866	17620	6.723	7461	5.440
*Mytilopsis leucophaeta*	800	0.478	701	0.485	1794	0.684	1055	0.769
*Dreissena polymorpha*	2686	1.605	1924	1.330	4872	1.859	2276	1.659
*Potamopyrgus antipodarum*	209	0.125	144	0.100	43807	16.714	23583	17.195
*Gillia altilis*	31795	19.003	25147	17.386	67309	25.681	32549	23.732
*Cipangopaludina chinensis*	2654	1.586	2024	1.399	5921	2.259	2876	2.097
*Melanoides tuberculata*	13	0.008	9	0.006	84	0.032	57	0.042
	**Trial Run**			
**Species**	**Read Numbers with FB**	**% Observed with FB**	**Read Number without FB**	**% Observed without FB**
*Dreissena polymorpha*	49883	64.312	29488	44.726
*Dreissena rostriformis*	2253	2.905	1449	2.198
*Sphaerium similie*	121	0.156	31	0.047
*Cipangopaludina chinensis*	0	0.000	0	0.000
*Sander vitreus*	11929	15.380	27159	41.194
*Corbicula fluminea*	12078	15.572	7164	10.867
*Potamopyrgus antipodarum*	19	0.024	5	0.008
*Valvata piscinalis*	992	1.279	205	0.311
*Sphaerium corneum*	18	0.023	6	0.009
*Mytilopsis leucophaeta*	0	0.000	0	0.000

### SPH16S assay mock communities

Results for the three samples (mock communities 1, 2, and 4) using the SPH16S primers show similar trends as found with the MOL16S assay, in that among the three sphaeriid species, observed read percentages reflected expected rank (Table 7). Furthermore, no reads from non-sphaeriid taxa were observed, indicating that this primer set is highly specific to sphaeriid clams.

**Table 7:** Number of reads, percentage observed and percentage expected for each of the mock community samples run with the SPH16S assay.

	Mock Community 1			Mock Community 2			Mock Community 4		
Species	Read Numbers	% Observed	% Expected	Read Numbers	% Observed	% Expected	Read Numbers	% Observed	% Expected
*Sphaerium similie*	108760	99.480	99.063	1461	1.437	16.645	500	0.006	3.196
*Dreissena rostriformis*	0	0.000	0.000	0	0.000	0.000	0	0.000	0.000
*Sander vitreus* (walleye)	0	0.000	0.000	0	0.000	0.000	0	0.000	0.000
*Sphaerium corneum*	551	0.504	0.785	16	0.016	0.128	82990	92.219	80.692
*Pisidium compressum*	17	0.016	0.153	100190	98.547	83.227	6502	7.225	16.112
*Mytilopsis leucophaeta*	0	0.000	0.000	0	0.000	0,000	0	0.000	0.000
*Dreissena polymorpha*	0	0.000	0.000	0	0.000	0.000	0	0.000	0.000
*Potamopyrgus antipodarum*	0	0.000	0.000	0	0.000	0.000	0	0.000	0.000
*Gillia altilis*	0	0.000	0.000	0	0.000	0.000	0.000	0.000	0.000
*Cipangopaludina chinensis*	0	0.000	0.000	0	0.000	0.000	0	0.000	0.000
*Melanoides tuberculata*	0	0.000	0.000	0	0.000	0.000	0	0.000	0.000

### eDNA aquaria samples

**Fig 3:**
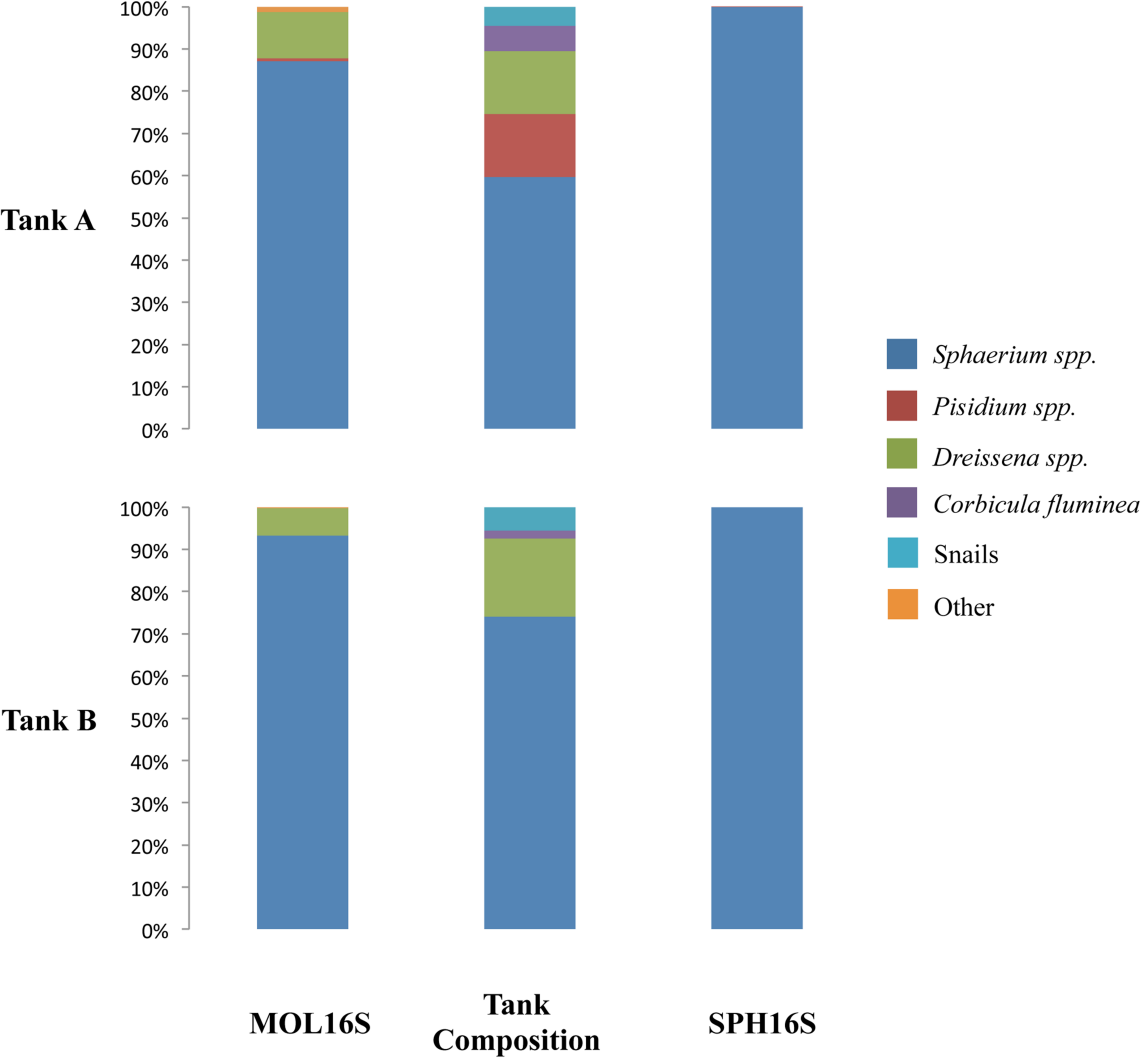
Bar charts denoting the proportion of reads for each assay (MOL16S and SPH16S) that were recovered from sequencing of water samples from tanks A and B. The middle bar shows the original composition of the tanks.

As expected, samples from both tanks run with the MOL16S primers yielded sequences comprising *D*. *polymorpha*, *D*. *rostriformis* and sphaeriid species. Based on known compositions within those tanks, sequence reads followed rank abundances, except for *C*. *fluminea* that did not amplify and the snails whose DNA did amplify but was not identified by our 97% threshold (Fig 3). The dominant sphaeriid species was *S*. *striatinum*, whereas *P*. *casertanum* and *P*. *compressum* also were discerned in tank A in which *Pisidum* clams had been placed. These three species were identified as common at the collection site (J. Boehler, pers. comm.). At the 97% similarity threshold, the MOL6S primers also identified a freshwater limpet (*Ferrissia fragilis*), oligochaete worms, bryozoans, and human DNA (Table 8). Although pleurocerid snails were placed in the tanks, no snail reads were identified at the 97% sequence similarity cut off. However, below this threshold, OTUs from three snail genera (*Leptoxis*, *Lithasia*, and *Pleurocera*) were detected (S6 Table). Given that pleurocerid species were identified as abundant at the collection site (J. Boehler, pers. comm.), it is likely that the actual species placed in the tanks belonged to these or other closely related snail genera, and that there is no sequence data for these specific species in GenBank. No *C*. *fluminea* were identified from the eDNA, although they had been placed in the tanks. Using the SPH16S assay, only the sphaeriid clams were identified at the 97% threshold (Fig 3) (Table 8).

**Table 8:** Number of reads per OTU identified at 97% similarity via BLAST search of aquaria samples.

Assay and Sample	Sequence ID Accession Number	Species	Number of Reads	Identity (%)	Coverage (%)	Percentage of total reads (%)	Percentage of just the 97% ID reads (%)
MOL16S Tank A	|gb|AF152045	*Sphaerium striatinum*	25707	98–99	100	8.989	86.737
	|gb|KP052744	*Dreissena polymorpha*	3037	100	100	1.062	10.247
	|gb|AF038996	*Dreissena rostriformis*	235	100	100	0.082	0.793
	|gb|KF889403	*Ferrissia fragilis*	218	100	100	0.076	0.736
	|gb|AY957830	**Pisidium casertanum	132	99	100	0.046	0.445
	|dbj|AB365626	*Plumatella emarginata*	117	100	100	0.041	0.395
	|gb|DQ459934	*Pristina aequiseta*	74	97–99	100	0.026	0.250
	|gb|JN681057	*Plumatella emarginata*	56	100	100	0.020	0.189
	|gb|AY957811	*Pisidium compressum*	52	99	100	0.018	0.175
	|gb|AF152044	*Sphaerium striatinum*	10	98	100	0.003	0.034
MOL16STank B	|gb|AF152045	*Sphaerium striatinum*	21806	99	100	7.922	93.128
	|gb|KP052744	*Dreissena polymorpha*	1286	100	100	0.467	5.492
	|gb|JX099457	*Dreissena rostriformis*	222	100	100	0.081	0.948
	|gb|KC429295	*Sphaerium nucleus	35	100	100	0.013	0.149
	|gb|JN681057	*Plumatella emarginata*	29	100	100	0.011	0.124
	|gb|GQ355403	*Chaetogaster diaphanus*	25	99	100	0.009	0.107
	|gb|KX594326	*Homo sapiens*	7	100	100	0.003	0.030
	|dbj|AB365626	*Plumatella emarginata*	5	100	100	0.002	0.021
SPH16STank A	|gb|AF152045	*Sphaerium striatinum*	240165	99	100	99.520	99.959
	|gb|AF152044	*Sphaerium striatinum*	416	98	98	0.172	0.173
	|gb|AY957830	**Pisidium casertanum	215	99	100	0.089	0.089
	|gb|KC429295	*Sphaerium nucleus	68	98	100	0.028	0.028
SPH16STank B	|gb|AF152045	*Sphaerium striatinum*	217725	99	100	99.783	99.978
	]gb|KC429295	*Sphaerium nucleus	48	100	100	0.022	0.022

*Denotes species from NCBI GenBank (https://www.ncbi.nlm.nih.gov/genbank/) not recognized on the World Register of Marine Species (WoRMS; http://www.marinespecies.org/).

** *Pisidium casertanum* is accepted as *Euglesa casertana*

### Maumee River field eDNA samples

**Fig 4:**
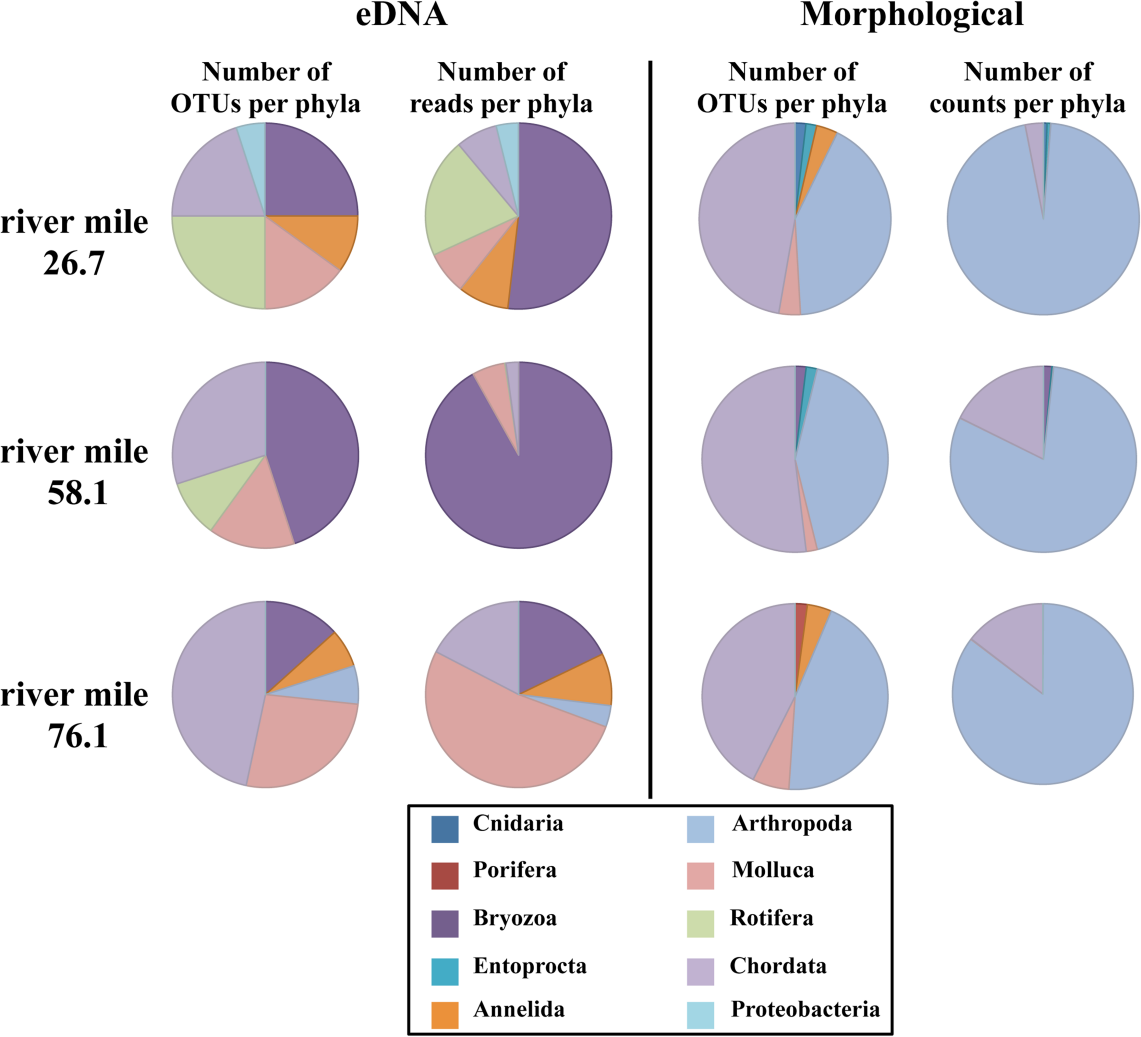
Pie charts comparing the molecular and morphological/ visual species identification methods from three different water samples taken on the Maumee River.

Comparing results from our metabarcoding assay to those from the Ohio EPA’s morphological identification, we find that the two approaches are biased to particular taxa. Given that we designed our MOL16S assay specifically for molluscs, this is not surprising; however, results show that some non-molluscan taxa including bryozoans, annelids, rotifers, and chordates also amplified. Without our fish blocking primer to reduce amplification of fish DNA, the number of fish reads also was not unexpected, however, we did not anticipate the amplification of those other taxa. Morphological identification of other taxonomic groups at the collection site was highest for fishes and arthropods (mainly insects) (S1 File). The morphological survey failed to discern sphaeriid clams or pleurocerid snails (from the *Physella* and *Elimia* genera) to species level, whereas our molecular assays identified them to species level (Table 9) (Fig 4). Interestingly the molecular assay found quagga mussel *D*. *rostriformis* in water samples at river miles 26.7 and 58.1, while the morphological survey did not record this species (Table 9). Furthermore, DNA from the European stream valve snail *Valvata piscninalis* was detected from river mile 58.1, but not detected visually in sampling. Some sequences were identified as belonging to the snail *Radix swinhoei* by our MOL16S molecular assay. This Asian species of snail has not been noted on watch lists as a potential invasive; however, upon further investigation including a BLAST search of the referenced OTU (GenBank accession number KP279638), the reference OTU sequence was closer to *Physella acuta* (a North American snail) than to other *Radix* species, indicating that the original *Radix swinhoei* GenBank entry (gb|KP279638) was incorrect. Thus we believe the correct identification of our reads for this OTU was *P*. *acuta*, which is a common snail species found in the sampling region, and not *R*. *swinhoei*. The morphological survey was able to discern one to three species of unionid mussels across the three water samples, whereas the MOL16S assay did not detect them. Finally, our water blank sample had 71 reads, eight of those being singletons with the remaining belonging to *Sphaerium striatinum* (Table 9), likely due to amplicon contamination in the lab.

**Table 9:** Number of reads per OTU identified at 97% similarity via BLAST search of Maumee River water samples.

Sample	Sequence ID Accession Number	Species	Number of Reads	Identity(%)	Coverage (%)	Percentage of total reads (%)	Percentage of the 97% ID reads (%)
Maumee River RM 26.7	|dbj|AB365626	*Plumatella emarginata*	476	98	100	0.064	49.739
	|gb|FJ426631	*Brachionus calyciflorus*	124	99	100	0.017	12.957
	|gb|HQ691208	*Aeolosoma sp*.	78	99	100	0.011	8.150
	|gb|GQ343273	*Brachionus calyciflorus*	47	100	100	0.006	4.911
	|gb|KX372733	*Homo sapiens*	43	100	100	0.006	4.493
	|gb|EU038309	*Physella acuta*	38	100	100	0.005	3.971
	|gb|CP012504	*Aeromonas veronii*	37	98	100	0.005	3.866
	|gb|AY093554	*Musculium transversum*	31	100	100	0.004	3.239
	|gb|FJ426630	*Brachionus angularis*	18	99	100	0.002	1.881
	|gb|KP306894	*Ictiobus cyprinellus*	14	100	100	0.002	1.463
	|dbj|AB365638	*Fredericella indica*	11	99	100	0.001	1.149
	|gb|JF275060	*Meleagris gallopavo*	8	100	100	0.001	0.836
	|gb|DQ459934	*Pristina aequiseta*	7	99	100	0.001	0.731
	|gb|GQ466406	*Brachionus calyciflorus*	7	98	91	0.001	0.731
	|dbj|AB365640	*Pectinatella magnifica*	5	100	100	0.001	0.522
	|gb|AY520093	*Aplodinotus grunniens*	4	100	100	0.001	0.418
	|gb|GQ343273	*Brachionus calyciflorus*	3	98	80	0.000	0.313
	|dbj|AB466325	*Paludicella articulata*	2	100	100	0.000	0.209
	|dbj|AB365642	*Lophopodella carteri*	2	100	100	0.000	0.209
	|gb|AF038996	*Dreissena rostriformis*	2	99	100	0.000	0.209
Maumee River RM 58.1	|dbj|AB365626	*Plumatella emarginata*	125320	97–100	98–100	14.204	65.371
	|dbj|AB365631	*Hyalinella punctata*	28959	97–98	100	3.282	15.106
	|dbj|AB365642	*Lophopodella carteri*	16787	98–100	100	1.903	8.757
	|gb|KJ186046	*Valvata piscinalis*	8101	100	100	0.918	4.226
	|dbj|AB365623	*Plumatella rugosa*	3236	100	100	0.367	1.688
	|gb|KP306894	*Ictiobus cyprinellus*	2744	100	100	0.311	1.431
	|gb|AF038996	*Dreissena rostriformis*	1760	98–99	100	0.199	0.918
	|dbj|AB365640	*Pectinatella magnifica*	1581	100	100	0.179	0.825
	|gb|DQ311116	*Elimia livescens*	1160	100	100	0.131	0.605
	|gb|KX372733	*Homo sapiens*	795	100	100	0.090	0.415
	|gb|AY216556	*Pimephales notatus*	391	97–100	94–100	0.044	0.204
	|gb|KM051966	*Brachionus angularis*	380	99	100	0.043	0.198
	|gb|GQ343301	*Plumatella emarginata*	140	97	100	0.016	0.073
	|dbj|AB126083	*Carpiodes carpio*	132	100	100	0.015	0.069
	|gb|AF038486	*Notropis atherinoides*	106	99	100	0.012	0.055
	|dbj|AB466325	*Paludicella articulata*	96	100	100	0.011	0.050
	|gb|GQ343296	*Plumatella emarginata*	7	98	99	0.001	0.004
	|dbj|AB365622	*Plumatella repens*	5	97	100	0.001	0.003
	|gb|KF162319	*Homo sapiens*	4	97	93	0.000	0.002
	|gb|FJ426630	*Brachionus angularis*	3	98	96	0.000	0.002
Maumee River RM 76.1	|gb|KP279638	*Radix swinhoei	903	100	100	0.255	24.048
	|dbj|AB365623	*Plumatella rugosa*	601	100	100	0.170	16.005
	|gb|AY093554	*Musculium transversum*	592	100	100	0.167	15.770
	|gb|DQ311116	*Elimia livescens*	427	100	100	0.121	11.372
	|gb|AY885589	*Pristina longiseta	341	99	100	0.096	9.081
	|gb|AY216556	*Pimephales notatus*	251	100	100	0.071	6.68
	|gb|FJ372631	*Saldula sp*.	140	98	100	0.040	3.73
	|gb|DQ536422	*Cyprinella spiloptera*	118	100	100	0.033	3.142
	|gb|KX353761	*Homo sapiens*	91	100	100	0.026	2.423
	|gb|KP013098	*Sander vitreus*	88	99	100	0.025	2.343
	|dbj|AB365628	*Plumatella reticulata*	69	100	100	0.019	1.838
	|gb|KR476977	*Sander lucioperca*	55	100	100	0.016	1.464
	|gb|DQ912062	*Dorosoma cepedianum*	34	100	100	0.010	0.905
	|gb|AF152045	*Sphaerium striatinum*	29	99	100	0.008	0.772
	|gb|KP013087	*Lepomis cyanellus*	16	100	100	0.005	0.426
Maumee River Blank	|gb|AF152044	*Sphaerium striatinum*	63	99	100		
	|dbj|AB365642	*Lophopodella carteri*	1	99	100		
	|dbj|AB365626	*Plumatella emarginata*	1	99	100		
	|gb|GQ343301	*Plumatella emarginata*	1	95	100		
	|gb|AF152044	*Sphaerium striatinum*	1	94	100		
	|dbj|AB365641	*Asajirella gelatinosa*	1	94	97		
	|gb|AF499051	*Synchaeta pectinata*	1	89	75		
	|gb|AF325131	*Asplanchna sieboldi	1	87	79		
	|gb|AF499051	*Synchaeta pectinata*	1	87	75		

* Denotes species from NCBI GenBank (https://www.ncbi.nlm.nih.gov/genbank/) not recognized in the World Register of Marine Species (WoRMS; http://www.marinespecies.org/).

## Discussion

### MOL16S assay with mock community samples

This study shows that careful primer design and testing reduces amplification bias in target amplicon sequencing, allowing for the inference of relative abundances among targeted taxa from high-throughput sequences of eDNA samples. Results from the mock communities support the hypothesis that the number of sequence reads correlates with the relative amounts of DNA initially put into the sample for each OTU. Although our MOL16S primer set is not specific to molluscs, it provides useful relative abundance measurements for the targeted group. The MOL16S primer set also amplifies some vertebrate and non-target invertebrate species. In fact after development of this marker, further research found that our forward MOL16S primer overlaps a previously designed primer used by Karlsson and Holmund [48] for forensic identification of mammal species. Thus we believe that this region of 16S may be very useful for development of eDNA assays that aim for species level identification. We also showed that our fish blocking primer successfully reduced the amplification of added walleye DNA in the mock communities. Interestingly, it was not reads from fishes that dominated our river samples as we expected, but rather reads from rotifers and bryozoans. Designing blocking primers for these taxa will decrease their amplification. Alternatively, this primer set may be very useful for efforts targeting these other taxa.

### SPH16S assay with mock community samples

Results from our SPH16S sphaeriid specific assay also demonstrated its ability to reflect relative abundance of the targeted species in a sample. Furthermore, and perhaps even more useful, these primers were shown to be extremely specific to the Sphaeriidae family, amplifying and identifying solely the target species in both the mock communities and the eDNA samples. Given the difficulty of morphological identification in this group, a concerted effort between sphaeriid taxonomists and molecular systematists would increase the amount of 16S sequence data from morphologically verified sphaeriid clams in genetic databases. Ultimately such an effort would enable biologists to then use the SPH16S marker as a mini-barcode for sphaeriid clams, thereby enhancing survey and monitoring efforts for this bivalve family.

### eDNA samples

Results from eDNA (water) samples with our two assays show an improvement over morphological surveys for bivalve and snail species identifications. In the aquaria trials, the MOL16S assay detected all bivalve taxa that were housed in the tank except for *C*. *fluminea*, which, at the time of water sampling, were buried in the tank substrate. We believe that any DNA shed by individuals of this species was less likely to enter the water column, thus reducing their detectability. Had we sampled the substrate, we likely would have had a better chance at detecting *C*. *fluminea*. Environmental DNA is not distributed homogenously in the environment, and species habitat preferences likely influence eDNA detection. Taking soil or substrate samples would be a better technique for detecting eDNA from benthic organisms. Snail species were not detected at the 97% similarity level, but several pleurocerid snail OTUs were identified just below that threshold. Despite North America having the highest diversity of snails in the family Pleuroceridae, current diversity and taxonomy of this group is not well understood [49–50] and like many other invertebrate taxa, few genetic data have been collected. We believe that this lack of pleurocerid sequences in GenBank led to our assays’ inability to make an identity at the 97% threshold.

Our SPH16S assay results demonstrate this primer pair’s specificity to sphaeriid bivalves. For both assays, the proportion of reads that aligned with *Sphaerium* spp. was higher than expected, especially in comparison to the *Dreissena* and *Pisidium* spp. Ten *Pisidium* and 10 *Dreissena* mussels were placed into tank A, but the number of reads assigned was much lower than expected. Individual *Pisidium* clams were much smaller in size than the *Sphaerium* clams, suggesting that biomass influences the amount of DNA detected. In fact other studies [7,51] report a strong relationship between biomass and eDNA, rather than between abundance (numbers of organisms) and eDNA. Biologists considering eDNA methods should first decide whether their question requires abundance as measured in number of individuals, or if biomass would suffice to answer their question.

The river water samples demonstrate that these metabarcoding assays improve species level identification for the targeted molluscan species relative to visual morphology. Our MOL16S assay detected three different snail and three different bivalve OTUs among the three water samples; whereas the visual inspection was unable to define the sphaeriid clams and pleurocerid snails to species level. Furthermore, our molecular assays detected invasive *D*. *rostriformis* and *V*. *piscinalis* that the visual method did not identify. The sole molluscan taxon that the visual method was able to discern to species was four unionid mussels. Upon further investigation of our molecular assay, we found several mismatches between our MOL16S primers and unionid mussel sequences from GenBank, thus explaining the lack of unionid sequences. Our MOL16S assay amplified several non-mollusc taxa as well, particularly bryozoans and rotifers. Given the abundance of these organisms in the aquatic communities, it is not surprising that most of the sequence reads aligned to these groups. Design of taxon specific primers to block these groups will likely improve the MOL16S assay’s ability to detect more mollusc eDNA. Although rotifers made up a smaller percentage of reads relative to the bryozoans at the 97% ID threshold, analysis of reads below that threshold (S7 Table) show that the majority of reads closely match a number of rotifer species. Our primers did not amplify many arthropods, whereas arthropods were one of the dominant groups that the morphological method diagnosed to species level.

Results from the Maumee River methods comparison demonstrate the respective strengths and weaknesses of both methods. Larger-sized taxa that are well studied such as insects (arthropods) and unionid mussels were readily diagnosed to species level through morphology by trained individuals. Our molecular assays outperformed this visual classification for other molluscs. The MOL16S assay also appears to be useful for bryozoan and rotifer identities. In fact the assay detected six genera and 10 different species of bryozoans, including the invasive *Lophopodella carteri*, whereas the morphological survey only diagnosed it and *Plumatella* spp. to the genus level. Given the diversity of bryozoans detected with our MOL16S assay, further work with this marker would aid assessment of its potential as a mini-barcode for bryozoans.

Finally our blank sample did suggest that some contamination occurred, but in a relatively small amount (Table 9). It is unclear as to whether the contamination occurred during the filtering or amplification stage. Given the multi-step PCR process employed for library preparation which included opening of tubes after amplification, it is likely that contamination could have occurred in the process. Nevertheless, the amount of contamination we observed was relatively small. Blanks should be used throughout the process in order to limit and document the extent of contamination.

Our study demonstrates that careful primer design is important in preserving relative abundance relationships among taxa in a metabarcoding study. This and our assays’ abilities to out preform morphological species level identification in less well studied taxa suggest that metabarcoding of eDNA samples will be a promising addition to biodiversity surveys. However one of the largest obstacles we came across when analyzing our field data was the lack of genetic data (and specifically voucher referenced data) in the publically-available genetic sequence databases. For example, our pleurocerid snail data from the aquaria experiments revealed that snail OTUs were only identified below the 97% similarity threshold. Thus, although we had snails of this family in the tanks, their DNA was not discerned according to our threshold. Similarly with the river water samples, a large number of rotifers were found below the 97% cut-off, thus not making our final species list. Finally, some of our reads showed 97% similarity to an OTU that was incorrectly identified in GenBank. For instance, some reads were identified by the BLAST search as *R*. *swinhoei* (gb|KP279638) and *Gammarus balcanicsus* (gb|DQ320034). Upon using these references in a BLAST (Basic Local Alignment Search Tool) search against the National Institutes of Health (NIH) NCBI (National Center for Biotechnology Information) nucleotide database (https://blast.ncbi.nlm.nih.gov/Blast.cgi), we found that the *R*. *swinhoei* sequence in GenBank (gb|KP279638) was more similar to *P*. *acuta* sequences than to other *Radix spp*. and the *G*. *balcanicsus* was more similar to insects than to other gammarid amphipods.

Checking suspicious results is important for data interpretation, especially when detection of invasive species can lead to rapid and costly management action. Researchers and managers interested in metabarcoding surveys need to be aware that, like any tool, metabarcoding comes with assumptions and limitations. For instance sequence divergence thresholds do not necessarily reflect actual species boundaries [52–53]. Furthermore the percentage similarity in sequence thresholds will vary with species, loci used, and the amount of genetic data available. Nevertheless, we believe that metabarcoding capabilities will be greatly improved with increase in taxonomically verified sequences from diverse taxa. As others have proposed, we emphasize the need for increased collaboration among taxonomists and molecular biologists to catalogue taxonomically verified specimens with genetic sequence barcode data across all taxa to increase the capability of genetic barcoding methods [54–57].

## Conclusions

Environmental DNA metabarcoding surveys can significantly augment efforts for identifying and eradicating invasive species, and conserving native species. Through low-impact sampling, eDNA samples processed with high-throughput sequencing can provide biological community composition data, identifying both native and invasive species. Our work shows that careful primer design and optimization can allow for targeted group analyses in which read abundance correlates well with original amount of DNA, providing potentially informative relative abundance or biomass data for taxa. We also have shown that eDNA methods and our two assays constitute useful tools for invasive species detection, as well as native mollusc (and perhaps other taxa) survey efforts, providing a valuable complement to traditional survey methods. The present study further demonstrates that metabarcoding data are only as good as the sequence and taxonomic information provided on genetic databases. Increased collaboration among taxonomists and molecular systematists is required in order to gain maximum benefits of this developing tool.

## Supporting information

S1 File Excel file of the Ohio EPA Maumee River sample data. (XLSX)

S1 Table List of targeted species for the Sphaeriidae SPH16S assay. * indicates invasive species whose extractions were used to test primers *in vitro*. Ŧ indicates native or non-invasive species also used to test primers. Parentheses indicate unique OTUs for the SPH16S amplicon, the number inside the parentheses represents the number of sequences belonging to that OTU.
(DOCX)

S2 Table Collection information for each of the samples used for the mock communities and primer design. (DOCX)

S3 Table Collection information for additional samples used for primer design. (DOCX)

S4 Table Number of reads, merged reads and exact match trimmed reads of the mock community samples. (DOCX)

S5 Table Number of reads, merged reads and processed reads of the eDNA samples. (DOCX)

S6 Table Number of reads per OTU identified below 97% similarity via BLAST search of aquaria samples. (DOCX)

S7 Table Number of reads per OTU identified below 97% similarity via BLAST search of Maumee River samples. (DOCX)
