# Supplementary material for: Environmental DNA (eDNA) metabarcoding assays to detect invasive invertebrate species in the Great Lakes
Source: PLoS One. 2017 May 18;12(5):e0177643. doi: 10.1371/journal.pone.0177643 (PMC5436814; doi:10.1371/journal.pone.0177643)
Supplement: S1 Table — * indicates invasive species whose extractions were used to test primers in vitro. Ŧ indicates native or non-invasive species also used to test primers. Parentheses indicate unique OTUs for the SPH16S amplicon, the number inside the parentheses represents the number of sequences belonging to that OTU. (DOCX) [file pone.0177643.s002.docx]

S1 Table. List of targeted species for the Spaeriidae SPH16S assay.  ***** indicates invasive species whose extractions were used to test primers *in vitro*. **Ŧ** indicates native or non-invasive species also used to test primers. Parentheses indicate unique OTUs for the SPH16S amplicon, the number inside the parentheses represents the number of sequences belonging to that OTU. **Ψ** denotes species from NCBI GenBank not recognized on the World Register of Marine Species (WoRMS). ** *Pisidium atkinsonium* and *P. casertanum* are recognized as *Euglesa atkinsoniana* and *E. casertana* on the World Register of Marine Species (WoRMS).

| *Scientific name* | **GenBank Accession number** |
| --- | --- |
| Ψ *Musculium argentinum* | (1) AF152034 |
| *M. indicum* | (1) KF483311 |
| Ψ *M. kashmirensis* | (2) KF483276–77 (10) KF483262–63, KF483308, KF483238–39, KF48324, KF483258, KF483246, AY093550, AY093553 |
| *M. lacustre* | (1) AY093552 (3) KF483289, AY093551, AF152035 |
| *M. partumeium* | (1) AF152036 |
| *M. securis* | (1) AF152033 |
| *M. transversum* | (1) AY093554 |
| *Pisidium adamsi* | (2) AY093556, AF152031 |
| ***P. atkinsonium (Euglesa atkinsoniana)* | (1) KF483307 (1) KF483313 |
| *P. amnicum* | (7) EU559086–89, AY093572, DQ062609–10 |
| *P. annandalei* | (1) EU559090 (1) KF483315 (1) KF483309 |
| ***P. casertanum (E. casertana)* | (1) AY957792 (1) AY093557 (1) \|AY093558 (1) AY093559 (2) AY957793–94 (4) AY957795–78 (2) AY957789–90 (2) EU559093–94 (1) EU559091 (1) EU559095 (2) EU559091–92 (1) EU559096 (1) EU559097 (1)\|AY957786 (2) AY957782–93 (11) AY957772-81, AY957830 (3) AY957784–85, AY957787 (1) AY957788 (3) DQ062613–15 (2) DQ062611, DQ062618 (1) DQ062612 (1) DQ062616 (1) DQ062617 (1) KF483293. (1) KF483316 (1) KF483290 (1) EU559098 (4) EU559099–101, KF483299 (1) EU559102 (1) KF483295 |
| Ψ *P. clarkeanum* | (1) EU559103 (1) EU559104 |
| _Ŧ_ *P. compressum* | (1) AY093560 (2) AF152029, KY426908 (1) AY957810 (1) AY957812 |
| Ψ *P. costulosum* | (1) KF483323 |
| Ψ *P. dammermani* | (1) KF483292 |
| *P. dubium* | (1) AF152027 |

| *Scientific name* | **GenBank Accession number** |
| --- | --- |
| Ψ *Pisidium edlaueri* | (1) EU559110 |
| *P. fallax* | (2) AY093561, AY957824 (2) AY957813. AY957868 |
| *P. ferrugineum* | (3) AY957884, AY957872–73 (8) AY957874– 80, AY957833 (1) AY957882 |
| Ψ *P.globulare* | (1) EU559114 |
| Ψ *P. hallae* | (1) AY093562 (1) AY957825 (5) AY957804, AY957806–07, AY957809, AY957832 (1) AY957805 (1) AY957808 |
| *P. henslowanum* | (12) DQ062620–22, DQ062645, EU559115–22 |
| Ψ *P. hibernicum* | (1) AY093563 |
| *P. insigne* | (1) AY957838 |
| Ψ *P. japonicum* | (1) AY093571 |
| *P. kuiperi* | (1) EU559123 (1) KF483306 (1) KF483310 |
| *P. lilljeborgi* | (2) AY957881, AY957839 (2) DQ062623–24 (2) KF483305, EU559124 |
| Ψ *P. maasseni* | (22) *P. maasseni*: KF483298, EU559125–29, EU559131, EU559130; *P. nitidium*: DQ062630–31, KF483317, EU559134–35; *P. edlaueri*: EU559106, EU559107–09,EU559111–13, KF483301–02 |
| *P. milium* | (13) AY0935664, AY957840–49, AF152028, DQ062625 (5) AY957850–54 (1) AY957855 |
| Ψ *P. nevillianum* | (2) EU559132–33 (1) KF483322 |
| Ψ *P. nipponense* | (1) AY093565 |
| *P. nitidium* | (1) AY093566 (2) AY957856, AY957858 (3) KF483285–86, KF483251 |
| *P. obtusale* | (1) EU559136 (2) EU559137–38 |

| *Scientific name* | **GenBank Accession** |
| --- | --- |
| Ψ *Pisidium parvum* | (1) AY093570 |
| *P. personatum* | (1) AY093567 (1) EU559139 (2) EU559140–41 (3) EU559142, EU559144, EU559146 (2) EU559143, EU559145 (2) DQ062632–33 (1) KF483288 (1) KF483318 |
| Ψ *P. pseudosphaerium* | (1) EU559147 |
| Ψ *P. sterkianum* | (1) AF152032 |
| Ψ *P. stewartii* | (29) KF483234–37, KF483240–244, KF483248–50, KF48354–57, KF48359–261, KF483264–68, KF483271, KF483273–74, KF483278–79 (1) KF483272 (1) KF483247 |
| *P. subtruncatum* | (2) EU559149, EU559154 (5) EU559150–53, EU559155 (1) \|EU559161 (1) EU559162 (1) KF483300 (1) DQ062636 (1) DQ062637 (2) DQ062643, AY093568 (3) KF483270, KF483280–81 (11) *P. subtruncatum*: KF483287, EU559156–60, DQ062638–39, DQ06264, KF483294 *P. henslowanum*: DQ062644 |
| *P. supinum* | (7) DQ062646–50, EU559148, AY093569 |
| Ψ *P. tenuilineatum* | (1) EU559163 |
| *P. variabile* | (2) AF152030, AY957799 (5) AY957800–803, AY957867 |
| *P. ventricosum* | (4) AY957834–37 |
| Ψ *P. viridarium* | (1) KF483296 |
| Ψ *P. waldeni* | (1) EU559164 |
| Ψ *P. zugmayeri* | (3) KF483275, KF483283–84 |
| **Sphaerium corneum* | (2) AY792318, AY792320 (3) GU128607, GU128610, GU128616 (1) GU12862 (31) GU128594–97, GU128599–604, GU128606, GU128611, GU128613–615, GU128617–625, GU128627–633 (8) AF152037, GU128598, GU128605, GU128608–09, GU128612, GU128634–35 (6) *S. corneum*: KY426905-06; *S. nucleus*: HM208271–73, KC429295 |
| *S. fabale* | (1) AF152039 |
| *S. novaezealandiae* | (1) AF152047 |

| *Scientific name* | **GenBank Accession number** |
| --- | --- |
| Ψ *Sphaerium nucleus* | (1) AY093573 (3) HM208267–69 (4) HM208264–66, HM208270 |
| *S. occidentale* | (1) AF152046 (1) DQ986373 |
| *S. rhomboideum* | (1) AF152038 (1) DQ986372 |
| Ψ *S. rivicola* | (3) HM014168–71 |
| *S. solidum* (accepted as S.striatinum) | (4) FJ874903–06 (1) FJ874907 (2) FJ874908–09 |
| _Ŧ_ *S. similie* | (1) KY426907 (1) AF152040 |
| *S.striatinum* | (1) AF152041 (1) AF152042 (1) AF152043 (1) AF152044 (1) AF152045 |
| Ψ *S. tasmanicum* | (1) AF152048 |
