# Supplementary material for: Environmental DNA (eDNA) metabarcoding assays to detect invasive invertebrate species in the Great Lakes
Source: PLoS One. 2017 May 18;12(5):e0177643. doi: 10.1371/journal.pone.0177643 (PMC5436814; doi:10.1371/journal.pone.0177643)
Supplement: S2 Table — (DOCX) [file pone.0177643.s003.docx]

S2 Table. Collections information for each of the samples used for the mock communities and primer design.

| **Species** | **Collection Year** | **Sampling Location** | **Collector/ ID** | **Accession Number** |  |
| --- | --- | --- | --- | --- | --- |
| *Sphaerium similie* | 2014 | Lost Creek, Northwest Ohio, USA | J. Boehler, K. Kreiger | KY426907 |  |
| *Dreissena rostriformis* | 1992 | Lake Ontario, Olcott, New York, USA | A. Spidle | KY426895 |  |
| *Sphaerium corneum* | 2013 | Gülper See, Gülpe, Brandenburg, Germany | C. Albrecht | KY426906 |  |
| *Pisidium compressum* | 2014 | Rock Creek, Tiffin, Ohio, USA | J. Boehler , K. Kreiger | KY426908 |  |
| *Mytilopsis leucophaeta* | 1997 | Bonnet Carré, Louisiana, USA | B. Thompson | KY426891 |  |
| *Dreissena polymorpha* | 2014 | Rhine River, Nierstein, Rhineland-Pfalz, Germany | C. Albrecht | KY426901 |  |
| *Potamopyrgus antipodarum* | 2014 | Black Earth Creek, Dane Co. Wisconsin, USA | T. Campbell | KY426909 |  |
| *Gillia altilis* | 2012 | North Carolina, USA | R. Dillon/R. Hershler | KY426912 |  |
| *Cipangopaludina chinensis* | 2009 | Big Bass Lake, Hubbard Co., Minnesota , USA | G. Montz | KY426911 |  |
| *Melanoides tuberculata* | 2008 | Nevada, USA | R. Hershler | KY426914 |  |
