# Supplementary material for: Environmental DNA (eDNA) metabarcoding assays to detect invasive invertebrate species in the Great Lakes
Source: PLoS One. 2017 May 18;12(5):e0177643. doi: 10.1371/journal.pone.0177643 (PMC5436814; doi:10.1371/journal.pone.0177643)
Supplement: S3 Table — (DOCX) [file pone.0177643.s004.docx]

S3 Table. Collections information for additional samples used for primer design.

| **Species** | **Collection Year** | **Sampling Location** | | | **Collector/ ID** | | **Accession Number** |
| --- | --- | --- | --- | --- | --- | --- | --- |
| *Dreissena rostriformis* | 1994 | Lake Erie, Fisherman Reef, Lorain, OH, USA | | | C. Stepien | | KY426892 |
| *Dreissena rostriformis* | 2001 | Rybinsk Resevoir, Volga River, Russia | | | I. Grigorovich | | KY426893 |
| *Dreissena rostriformis* | 2002 | S. Dneiper River, Kherson, Ukraine | |  | I. Grigorovich | | KY426894 |
| *Dreissena rostriformis* | 2014 | Main River, Steinheim, Hessen, Germany |  | | C. Albrecht |  | KY426896 |
| *Dreissena polymorpha* | 1995 | Hudson River, NY, USA | | | D. Strayer | | KY426897 |
| *Dreissena polymorpha* | 2014 | w. Lake Erie, Oregon ,OH, USA | | | N. Marshall | | KY426898 |
| *Dreissena polymorpha* | 2014 | w. Lake Erie, Oregon, OH, USA | | | N. Marshall | | KY426899 |
| *Dreissena polymorpha* | 1992 | Lake Ontario, Olcott, NY, USA | | | A. Spindle | | KY426900 |
| *Dreissena polymorpha* | 1993 | Lake IJsselmeer, Netherlands | | | H. Jenner | | KY426902 |
| *Corbicula fluminea* | 1997 | Mohican River, OH, USA | | | C. Stepien | | KY426903 |
| *Corbicula fluminea* | 2000 | Mohican River, OH, USA | | | C. Stepien | | KY426904 |
| *Sphaerium corneum* | 2013 | Rhine River, Gülpe, Brandenburg, Germany | | | C. Albrecht | | KY426905 |
| *Potamopyrgus antiposarum* | 2014 | Black Earth Creek, Dane Co. WI, USA | | | T. Campbell | | KY426909 |
| *Radix balthica* | 2014 | Imsbach, Linden, Heesen, Germany | | | C. Albrecht | | KY426913 |
