# Supplementary material for: Environmental DNA (eDNA) metabarcoding assays to detect invasive invertebrate species in the Great Lakes
Source: PLoS One. 2017 May 18;12(5):e0177643. doi: 10.1371/journal.pone.0177643 (PMC5436814; doi:10.1371/journal.pone.0177643)
Supplement: S4 Table — (DOCX) [file pone.0177643.s005.docx]

S4 Table. Number of reads, merged reads and exact match trimmed reads of the mock community samples.

| **Sample** | **Mock Community** | **Assay** | **Number of Reads** | **Number of Merged Reads** | **% Reads Merged** | **Number of Reads Trimmed and Exact Match** | **% Reads Trimmed and Exact Match** |
| --- | --- | --- | --- | --- | --- | --- | --- |
|  |  |  |  |  |  |  |  |
| SC3Pro1 | 1 | MOL16S with Fish Blocking Primer | 689712 | 639530 | 92.72 | 320292 | 50.08 |
| SC3Pro2 | 2 | MOL16S with Fish Blocking Primer | 405048 | 358270 | 88.45 | 173895 | 48.54 |
| SC3Pro3 | 3 | MOL16S with Fish Blocking Primer | 402219 | 370617 | 92.14 | 167315 | 45.14 |
| SC3Pro4 | 4 | MOL16S with Fish Blocking Primer | 671241 | 608080 | 90.59 | 262097 | 43.10 |
| SC3Pro5 | 5 | MOL16S with Fish Blocking Primer | 480606 | 441675 | 91.90 | 208961 | 47.31 |
| NFSC3Pro3 | 3 | MOL16S without Fish Blocking Primer | 349590 | 320799 | 91.76 | 144638 | 45.09 |
| NFSC3Pro4 | 4 | MOL16S without Fish Blocking Primer | 420015 | 337195 | 80.28 | 137150 | 40.67 |
| SPSC3Pro1 | 1 | SPH16S | 425271 | 372072 | 87.49 | 109328 | 29.38 |
| SPSC3Pro2 | 2 | SPH16S | 341476 | 293789 | 86.04 | 101667 | 34.61 |
| SPSC3Pro4 | 4 | SPH16S | 410780 | 348335 | 84.80 | 89992 | 25.83 |
