# Supplementary material for: Environmental DNA (eDNA) metabarcoding assays to detect invasive invertebrate species in the Great Lakes
Source: PLoS One. 2017 May 18;12(5):e0177643. doi: 10.1371/journal.pone.0177643 (PMC5436814; doi:10.1371/journal.pone.0177643)
Supplement: S5 Table — (DOCX) [file pone.0177643.s006.docx]

S5 Table. Number of reads, merged reads and processed reads of the eDNA samples.

| **Experiment** | **Assay** | **Number of Reads** | **Number of Merged Reads** | **% of Merged Reads** | **Number of Reads Processed** | **% of Reads Processed** | **Number of Reads Assigned an OTU** | **% of Reads Assigned an OTU** | **Number of Reads Assigned an OTU at 97% ID** | **% of Reads Assigned an OTU at 97% ID** |
| --- | --- | --- | --- | --- | --- | --- | --- | --- | --- | --- |
| Aquaria-Tank A | MOL16S | 306311 | 300789 | 98.20 | 285980 | 95.08 | 52846 | 18.48 | 29638 | 10.36 |
| Aquaria-Tank A | SPH16S | 498926 | 482018 | 96.61 | 241323 | 50.07 | 241313 | 99.99 | 240264 | 99.56 |
| Aquaria- Tank B | MOL16S | 291954 | 287172 | 98.36 | 275268 | 95.86 | 23780 | 8.64 | 23415 | 8.51 |
| Aquaria- Tank B | SPH16S | 240360 | 236332 | 98.32 | 218198 | 92.33 | 218187 | 99.99 | 217773 | 99.81 |
| Maumee River RM 26.7 | MOL16S | 821849 | 777282 | 94.58 | 742136 | 95.48 | 661880 | 89.19 | 957 | 0.13 |
| Maumee River RM 58.1 | MOL16S | 1119271 | 1087219 | 97.14 | 882293 | 81.15 | 773198 | 87.64 | 191707 | 21.73 |
| Maumee River RM 76.1 | MOL16S | 709472 | 680864 | 95.97 | 353983 | 51.99 | 275917 | 77.95 | 3755 | 1.06 |
| Maumee River Blank | MOL16S | 2433 | 118 | 4.85 | 63 | 53.39 | 63 | 100.00 | 63 | 100.00 |
