# Supplementary material for: Environmental DNA (eDNA) metabarcoding assays to detect invasive invertebrate species in the Great Lakes
Source: PLoS One. 2017 May 18;12(5):e0177643. doi: 10.1371/journal.pone.0177643 (PMC5436814; doi:10.1371/journal.pone.0177643)
Supplement: S6 Table — (DOCX) [file pone.0177643.s007.docx]

S6 Table. Number of reads per OTU identified below 97% similarity via BLAST search of aquaria samples. ***** denotes species from NCBI GenBank not recognized on the World Register of Marine Species (WoRMS).

| **Assay and Sample** | **Sequence ID Accession Number** | **Species** | **Number of Reads** | **Percent Identity** | **Organism** |
| --- | --- | --- | --- | --- | --- |
| MOL16S  TankA | \|gb\|HQ691208 | *Aeolosoma sp.* | 3 | 87 | oligochaete worm |
|  | \|gb\|CP007518 | *Aeromonas hydrophila* | 116 | 88 | proteobacteria |
|  | \|gb\|AF325131 | ********Asplanchna sieboldi* | 2 | 83 | rotifer |
|  | \|gb\|JN035719 | *Brachionus plicatilis* | 2 | 85 | rotifer |
|  | \|gb\|KP063117 | *Cypridopsis vidua* | 18 | 89 | ostracod- crustacean |
|  | \|gb\|KP052744 | *Dreissena polymorpha* | 41 | 96 | zebra mussel |
|  | \|gb\|JX099457 | *Dreissena rostriformis* | 18 | 95 | quagga mussel |
|  | \|gb\|AF101000 | *Leptoxis virgata* | 41 | 89 | pleurocerid snail |
|  | \|gb\|AF100998 | *Lithasia armigera* | 12 | 92 | pleurocerid snail |
|  | \|emb\|FR856884 | *Philodina citrina* | 106 | 83-93 | rotifer |
|  | \|gb\|KT164312 | *Pleurocera prasinata* | 67 | 92 | pleurocerid snail |
|  | \|gb\|KT164365 | *Pleurocera pyrenella* | 16 | 90 | pleurocerid snail |
|  | \|gb\|DQ459934 | *Pristina aequiseta* | 14 | 96 | oligochaete worm |
|  | \|gb\|GQ355417 | *Pristina osborni* | 21546 | 95 | oligochaete worm |
|  | \|gb\|GQ304898 | *Rotaria rotatoria* | 65 | 83 | rotifer |
|  | \|gb\|AF152045 | *Sphaerium striatinum* | 35 | 95 | sphaeriid mussel |
| MOL16S  Tank B | \|gb\|HQ691208 | *Aeolosoma sp.* | 144 | 87 | oligochaete worm |
|  | \|gb\|GQ355404 | *Chaetogaster diastrophus* | 12 | 96 | oligochaete worm |
|  | \|gb\|KP063117 | *Cypridopsis vidua* | 13 | 89-91 | ostracod- crustacean |
|  | \|gb\|KP052744 | *Dreissena polymorpha* | 25 | 96 | zebra mussel |
|  | \|gb\|JX099457 | *Dreissena rostriformis* | 17 | 95 | quagga mussel |
|  | \|gb\|KP965862 | *Lepidodermella squamata* | 2 | 94 | gastrotrich |
|  | \|gb\|AF101000 | *Leptoxis virgata* | 14 | 89 | pleurocerid snail |
|  | \|emb\|FR856884 | *Philodina citrina* | 8 | 88 | rotifer |
|  | \|gb\|GQ387649 | *Rhopalomyia pomum* | 2 | 81 | insect |
|  | \|gb\|GU128616 | *Sphaerium corneum* | 6 | 96 | sphaeriid mussel |
|  | \|gb\|KC429295 | *Sphaerium nucleus* | 2 | 96 | sphaeriid mussel |
|  | \|gb\|AF152045 | *Sphaerium striatinum* | 24 | 94-96 | sphaeriid mussel |
| SPH16S  Tank A | \|gb\|CP015448 | *Aeromonas veronii* | 15 | 95 | proteobacteria |
|  | \|gb\|HQ912524 | *Cyclophora tenuis* | 25 | 82 | diatom |
|  | \|gb\|AF152036 | *Musculium partumeium* | 4 | 96 | sphaeriid mussel |
|  | \|gb\|AY957830 | *Pisidium casertanum* | 5 | 95 | sphaeriid mussel |
|  | \|gb\|DQ062616 | *Pisidium casertanum* | 15 | 96 | sphaeriid mussel |
|  | \|gb\|EU559137 | *Pisidium obtusale* | 3 | 95 | sphaeriid mussel |
|  | \|gb\|AF152046 | *Sphaerium striatinum* | 50 | 95 | sphaeriid mussel |
|  | \|gb\|AF152045 | *Sphaerium striatinum* | 218 | 88-96 | sphaeriid mussel |
|  | \|gb\|AF152044 | *Sphaerium striatinum* | 13 | 93-96 | sphaeriid mussel |
|  | \|gb\|AF152043 | *Sphaerium striatinum* | 52 | 96 | sphaeriid mussel |
|  | \|gb\|AF152041 | *Sphaerium striatinum* | 2 | 96 | sphaeriid mussel |
| SPH16S  Tank B | \|gb\|DQ311122 | *Leptoxis dilatata* | 2 | 89 | pleurocerid snail |
|  | \|gb\|AF152036 | *Musculium partumeium* | 3 | 96 | sphaeriid mussel |
|  | \|gb\|KC429295 | ********Sphaerium nucleus* | 2 | 96 | sphaeriid mussel |
|  | \|gb\|AF152046 | *Sphaerium occidentale* | 135 | 95 | sphaeriid mussel |
|  | \|gb\|AF152045 | *Sphaerium striatinum* | 182 | 92-96 | sphaeriid mussel |
