# Supplementary material for: Environmental DNA (eDNA) metabarcoding assays to detect invasive invertebrate species in the Great Lakes
Source: PLoS One. 2017 May 18;12(5):e0177643. doi: 10.1371/journal.pone.0177643 (PMC5436814; doi:10.1371/journal.pone.0177643)
Supplement: S7 Table — (DOCX) [file pone.0177643.s008.docx]

S7 Table. Number of reads per OTU identified below 97% similarity via BLAST search of Maumee River samples. ***** denotes species from NCBI GenBank not recognized on the World Register of Marine Species (WoRMS).

| **Sample** | **Sequence ID Accession Number** | **Species** | **Number of Reads** | **Percent Identity** | **Organism** |
| --- | --- | --- | --- | --- | --- |
| Maumee River RM 26.7 | \|gb\|AF325131 | ******Asplanchna sieboldi* | 640709 | 78-94 | rotifer |
|  | \|gb\|FJ426630 | *Brachionus angularis* | 28 | 92 | rotifer |
|  | \|gb\|FJ230772.1 | *Brachionus calyciflorus* | 4 | 94 | rotifer |
|  | \|gb\|GQ466406 | *Brachionus calyciflorus* | 3 | 96 | rotifer |
|  | \|gb\|GQ343273 | *Brachionus calyciflorus* | 29 | 87-96 | rotifer |
|  | \|gb\|GQ203146 | *Brachionus calyciflorus* | 4 | 87-93 | rotifer |
|  | \|gb\|GQ203181 | *Brachionus calyciflorus* | 14 | 93 | rotifer |
|  | \|gb\|FJ426632 | *Brachionus caudatus* | 75 | 85-94 | rotifer |
|  | \|gb\|KM051964 | ******Brachionus dimidiatus* | 83 | 83-85 | rotifer |
|  | \|gb\|\|JN035841 | *Brachionus manjavacas* | 2 | 86 | rotifer |
|  | \|gb\|JN035779 | *Brachionus manjavacas* | 86 | 82-85 | rotifer |
|  | \|gb\|JN035819 | *Brachionus manjavacas* | 18 | 83-85 | rotifer |
|  | \|gb\|AF325132 | *Brachionus patulus* | 71 | 82 | rotifer |
|  | \|gb\|JN035719 | *Brachionus plicatilis* | 35 | 85-86 | rotifer |
|  | \|emb\|AM180757 | *Brachionus plicatilis* | 49 | 86 | rotifer |
|  | \|emb\|AM180757 | *Brachionus plicatilis* | 20 | 86-89 | rotifer |
|  | \|gb\|FJ426635 | *Brachionus quadridentatus* | 2 | 92 | rotifer |
|  | \|gb\|KJ489417 | *Brachionus rubens* | 86 | 85 | rotifer |
|  | \|gb\|GQ894755 | *Brachionus sp.* | 3 | 86 | rotifer |
|  | \|gb\|GQ355404 | *Chaetogaster diastrophus* | 16 | 97 | oligochaete worm |
|  | \|gb\|AF456851 | *Chironomus sp.* | 2 | 91 | insect |
|  | \|emb\|LT578417 | *Cyanobium sp* | 2 | 89 | cyanobacteria |
|  | \|gb\|DQ320034. | *Gammarus balcanicus*** | 13 | 85-86 | **misidentified |
|  | \|dbj\|AB365631 | *Hyalinella punctata* | 40 | 96.5 | bryozoan |
|  | \|gb\|KP965862 | *Lepidodermella squamata* | 2 | 92 | gastrotrich |
|  | \|gb\|FR856884 | *Philodina citrina* | 7 | 87 | rotifer |
|  | \|gb\|KT164312 | *Pleurocera prasinata* | 18 | 91 | Pleurocerid snail |
|  | \|dbj\|AB365626 | *Plumatella emarginata* | 9 | 95 | bryozoan |
|  | \|dbj\|AB365628 | *Plumatella reticulata* | 31 | 95 | bryozoan |
|  | \|gb\|\|AF499051 | *Synchaeta pectinata* | 4 | 86 | rotifer |
| Maumee River RM 58.1 | \|dbj\|AB365641 | *Asajirella gelatinosa* | 1345 | 93 | bryozoan |
|  | \|gb\|AF325131 | ******Asplanchna sieboldi* | 399162 | 80-90 | rotifer |
|  | \|gb\|FJ426630 | *Brachionus angularis* | 330 | 92-93 | rotifer |
|  | \|gb\|EU719112 | *Brachionus calyciflorus* | 73 | 80 | rotifer |
|  | \|gb\|FJ230774 | *Brachionus calyciflorus* | 2 | 93 | rotifer |
|  | \|gb\|GQ343273 | *Brachionus calyciflorus* | 366 | 96 | rotifer |
|  | \|gb\|FJ426632 | *Brachionus caudatus* | 3790 | 85-95 | rotifer |
|  | \|gb\|KM051964 | *Brachionus dimidiatus* | 2 | 83 | rotifer |
|  | \|gb\|JN035779 | *Brachionus manjavacas* | 7921 | 82-83 | rotifer |
|  | \|gb\|AF325132 | *Brachionus patulus* | 1129 | 81 | rotifer |
|  | \|emb\|AM180759 | *Brachionus plicatilis* | 8 | 84 | rotifer |
|  | \|emb\|AM180757 | *Brachionus plicatilis* | 2 | 88 | rotifer |
|  | \|gb\|GQ894755 | *Brachionus sp.* | 12 | 85-86 | rotifer |
|  | \|gb\|DQ320034 | *Gammarus balcanicus *** | 468 | 85 | misidentified ** |
|  | \|dbj\|AB365631 | *Hyalinella punctata* | 2226 | 94 | bryozoan |
|  | \|gb\|KP306894 | *Ictiobus cyprinellus* | 2 | 96 | fish |
|  | \|dbj\|AB365642 | *Lophopodella carteri* | 41 | 95-96 | bryozoan |
|  | \|dbj\|AB365629 | *Plumatella casmiana* | 1387 | 95 | bryozoan |
|  | \|gb\|GQ343296 | *Plumatella emarginata* | 3422 | 93-96 | bryozoan |
| Maumee River RM 76.1 | \|gb\|FJ561294 | *Aeromonas sp* | 21 | 96 | bacteria |
|  | \|gb\|CP015005 | *Aminobacter aminovorans* | 53 | 85 | bacteria |
|  | \|gb\|AF325131 | ******Asplanchna sieboldi* | 146213 | 79-89 | rotifer |
|  | \|gb\|KM051966 | *Brachionus angularis* | 76 | 86 | rotifer |
|  | \|gb\|FJ426630 | *Brachionus angularis* | 5 | 86-87 | rotifer |
|  | \|gb\|EU719112 | *Brachionus calyciflorus* | 13 | 81-86 | rotifer |
|  | \|gb\|FJ230774 | *Brachionus calyciflorus* | 888 | 85-95 | rotifer |
|  | \|gb\|GQ466406 | *Brachionus calyciflorus* | 26 | 80-81 | rotifer |
|  | \|gb\|GQ343273 | *Brachionus calyciflorus* | 4 | 88 | rotifer |
|  | \|gb\|FJ426632 | *Brachionus caudatus* | 10 | 84-88 | rotifer |
|  | \|gb\|JN035841 | *Brachionus manjavacas* | 17 | 83-86 | rotifer |
|  | \|gb\|JN035779 | *Brachionus manjavacas* | 841 | 83 | rotifer |
|  | \|gb\|JN035819 | *Brachionus manjavacas* | 127 | 83-88 | rotifer |
|  | \|gb\|AF325132 | *Brachionus patulus* | 19592 | 80-84 | rotifer |
|  | \|gb\|JN035719 | *Brachionus plicatilis* | 43125 | 86 | rotifer |
|  | \|dbj\|AP009407 | *Brachionus plicatilis* | 71 | 89 | rotifer |
|  | \|emb\|AJ748693 | *Brachionus plicatilis* | 2893 | 83-87 | rotifer |
|  | \|emb\|AM180757 | *Brachionus plicatilis* | 20 | 84 | rotifer |
|  | \|gb\|KJ489417 | *Brachionus rubens* | 165 | 81-85 | rotifer |
|  | \|gb\|GQ894755 | *Brachionus sp.* | 5 | 82-83 | rotifer |
|  | \|gb\|DQ459957 | *Branchiura sowerbyi* | 1298 | 88 | oligochaete worm |
|  | \|gb\|GQ355404 | *Chaetogaster diastrophus* | 178 | 96 | oligochaete worm |
|  | \|gb\|AF456851 | *Chironomus sp.* | 21 | 91 | insect |
|  | \|gb\|KC177440 | ******Chironomus tepperi* | 17 | 91 | insect |
|  | \|emb\|LT578417 | *Cyanobium sp* | 18 | 96 | cyanobacteria |
|  | \|gb\|KT956326 | ******Homoneura biumbrata* | 25 | 88 | insect |
|  | \|gb\|KP965862 | *Lepidodermella squamata* | 56 | 93 | gastrotrich |
|  | \|gb\|CP011773 | *Mycobacterium sp.* | 35 | 91 | bacteria |
|  | \|gb\|GQ304898 | *Rotaria rotatoria* | 4198 | 89 | rotifer |
|  | \|gb\|DQ266474 | *Stenelmis sp.* | 42 | 91 | insect |
|  | \|gb\|AY250946 | ******Stictotarsus bertrandi* | 5 | 82 | insect |
|  | \|gb\|CP010904 | *Verrucomicrobia bacterium* | 99 | 83 | bacteria |
